# AI-driven antimicrobial peptide characterization unveils novel motifs for drug design

**DOI:** 10.1038/s41598-025-30419-1

**Published:** 2025-12-29

**Authors:** Sarala Padi, Kinjal Mondal, David P. Hoogerheide, Frank Heinrich, Mihaela Mihailescu, Jeffery B. Klauda, Antonio Cardone

**Affiliations:** 1 Information Technology Laboratory (ITL), NIST, Gaithersburg, MD 20899 USA; 2 NIST Center for Neutron Research (NCNR), NIST, Gaithersburg, MD 20899 USA; 3 Institute for Bioscience and Biotechnology Research (IBBR), UMD, Rockville, MD 20850 USA; 4 Department of Physics, Carnegie Mellon University, Pittsburgh, PA 15213 USA; 5 Institute for Physical Science and Technology, Biophysics Program, UMD, College Park, MD 20742 USA; 6 Department of Chemical and Biomolecular Engineering, UMD, College Park, MD 20742 USA

**Keywords:** Antimicrobial resistance, Antimicrobial peptides, Drug design, Motif extraction, Topic model, Minimum inhibitory concentration, Computational biology and bioinformatics, Drug discovery, Microbiology

## Abstract

Antibiotics have been developed to effectively target and eliminate bacteria, but the rise in antimicrobial resistance (AR) complicates the treatment of certain infections. To address this issue, researchers have explored antimicrobial peptides (AMPs) that disrupt bacterial membranes. A promising method for this exploration is motif-based analysis, which identifies hidden patterns in AMPs to better understand their mechanism of action. While existing methods rely on expert knowledge, incorporating topic models can enhance analysis by revealing the contextual relationships between sequence elements. This is complemented by a data analytics tool designed to analyze AMP motifs and their biochemical properties. Such integration allows for the extraction of valuable motifs and the development of a robust data analytics module for predicting membrane activity. Additionally, we evaluated the biological relevance of motifs by extracting biochemical features, making structural predictions via Evolutionary Scale Modeling (ESM). Our results indicate that topic model-derived motifs are strongly associated with antimicrobial activity and demonstrate lower minimum inhibitory concentration values and capture contextual information more effectively than traditional frequency-based motifs. We also performed a comparative analysis between the two approaches regarding motif evolution, sequence-level attributes, and entropy measures, ultimately contributing to ongoing efforts to combat AR.

## Introduction

Antibiotics have been developed to treat bacterial infections, significantly improving health and life expectancy. However, some bacterial strains develop resistance to antibiotics, known as antibacterial resistance (AR)^1^. AR increases the risk of severe illness and poses a global public health threat, causing millions of deaths annually^2–4^. In response, the World Health Assembly adopted a global action plan to combat AR by investing in new medicines and interventions^5^.

To effectively combat infections caused by AR, scientists are focusing on antimicrobial peptides (AMPs) as promising candidates for the next generation of antibiotics^6,7^. AMPs, naturally found in all living organisms, play a crucial role in defending against fungi, viruses, and bacteria^8–11^. These peptides, consisting of 10 to 60 amino acids, can be engineered to disrupt bacterial cell membranes^9^. The effectiveness of AMPs relies on their unique biochemical properties, particularly the arrangement of structural features like $\alpha$-helices and $\beta$-sheets. To advance AMP research, a strong interdisciplinary approach that integrates biology, material science, chemistry, and bioinformatics is essential. Such collaboration is vital for developing innovative solutions to combat AR^6,8,9,12^.

Motifs are short subsequences linked to secondary structures, like helices. Identifying new sequences often shows limited similarities to known ones, emphasizing the importance of motif analysis for understanding their roles^13^. By characterizing motifs, we gain insights into peptide functionality, particularly when structurally distinct peptides serve similar functions. They play crucial roles in biological processes such as gene expression regulation, protein design, and functional annotation^14–16^. Although regular expressions are often used for motif identification, they require prior knowledge and may miss important motifs. To improve motif discovery, complementary approaches like statistical models can be helpful. This study uses topic models to automatically extract motifs from protein sequences, enhancing our understanding of protein functionality.

Recent advances in computational biology have led to notable progress in the development of AI models, including large language models, such as ESM^17^, ProGen^18^, and ProtBERT^19^, as well as structure prediction models, including AlphaFold^20^ and ESMfold^17^. Although these models excel in function, sequence generation, and structure prediction, their reliance on extensive datasets for fine-tuning can limit adaptability and the extraction of meaningful insights. Additionally, these methods can be biased, particularly in recognizing novel motifs that are less represented in the data. Inspired by techniques from natural language processing, topic models effectively reveal hidden structures and relationships without labeled examples, offering better interpretability than traditional protein-based models^21^. Previous studies have shown that topic models provide deeper insight into protein-protein interactions, identify meaningful patterns, and facilitate a deeper understanding of biological systems^22–24^.

Several computational approaches have been proposed for motif discovery, ranging from traditional sequence-based algorithms to AI-driven frameworks, but each has limitations when it comes to AMPs. For example, AMPlify^25^ highlights key residues using attention mechanisms but does not define functional motifs. Similarly, MotifQuest^26^ and Gemoda^27^ , while useful for mass spectrometry data and k-mer clustering, lack the contextual understanding needed for AMP analysis.

In DNA motif discovery, algorithms like MEME, Weeder, and AlignACE have been validated on synthetic datasets with known motifs, enabling clear benchmarking^28^. However, the absence of comparable ground truth motifs for AMPs, which derive their functional motifs from sequence activity, presents an opportunity for developing tailored benchmarking approaches. The BML web server^29^ and AI frameworks like PLPTP^30^ offer insights into motif discovery, yet they tend to focus on regulatory DNA sequences and toxicity prediction, limiting their application to AMPs. This highlights a key challenge: the lack of established ground truths for biologically significant motifs complicates the assessment of their functional relevance. Similarly, machine learning–based AMP generation pipelines often rely on extracting recurring motifs from sequences predicted as antimicrobial. These motifs are then ranked by frequency or statistical association (e.g., lasso regression) and used as building blocks for new peptide design^31^. While this provides interpretability, the approach treats motifs as isolated fragments and ignores the broader sequence context.

To address these gaps, we propose a topic modeling framework that treats motifs as latent themes within AMP sequences, providing a more context-aware representation. This approach allows for the discovery of coherent patterns critical for antimicrobial activity. Our AI-driven framework designed to extract meaningful motifs from AMPs by dividing sequences into smaller subsequences or k-mers. This thematic approach aids in revealing critical patterns essential for antimicrobial activity.

Furthermore, we develop a data analysis framework to highlight key motifs. These motifs represent promising candidates for AMP design and exhibit functional diversity across various thematic clusters. Our analysis identifies motifs and computes properties to improve biological understanding by mapping sequence level properties to motifs and computing motif level properties.

We develop a data analysis framework that highlights key motifs, which are promising candidates for AMP design. These motifs show functional diversity across different thematic clusters and enhance biological understanding by linking sequence properties to motifs and assessing their characteristics. We investigate how motif sizes and different topic counts affect motif extraction and data clarity. Our findings indicate that motifs derived from topic models are more diverse and capture contextual information more effectively than those based on frequency from databases. Additionally, we compared both types of motifs in terms of their relevance to motif evolution, sequence attributes, and entropy measures. This comprehensive approach enhances the understanding of antimicrobial research and contributes positively to tackling antimicrobial resistance.

## Methods

The identification of hidden patterns within antimicrobial peptide sequences offers significant opportunities for understanding their structural functionalities and mechanisms of action, particularly in terms of membrane binding, insertion, and disruption. These patterns or subsequences represent critical structural elements that are essential for the biological activity of AMPs. However, identifying such subsequences is inherently complex because of the vast and diverse range of AMPs available in current biological databases. In many cases the identification process relies heavily on the expertise of domain specialists who manually examine the sequences, drawing on their knowledge of specific AMP classes and associated biophysical properties.

We propose a framework for motif analysis in AMPs, focusing on k-mers—short subsequences crucial for antimicrobial activity. Our framework includes three modules: (1) encoding or representing AMPs, (2) using topic models to generate distributions, and (3) mapping extracted motifs to relevant properties. This structured approach aims to uncover the biological significance of identified motifs. We adapt Latent Dirichlet Allocation (LDA), a proven model in natural language processing, for motif discovery in biological sequences. By developing a pipeline that applies LDA to AMP sequences and validating the motifs, we can reveal biologically relevant patterns and enhance our understanding of membrane activity. This work extends the application of LDA and opens new research avenues in the field of antimicrobial peptides.

### Encoding of AMPs: k-mer generation

**Fig. 1:**
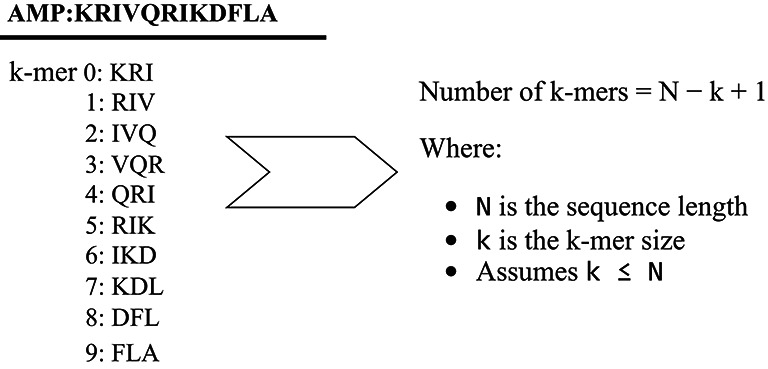
An illustration of k-mer generation for a given sequence with a length of 12 and a k-mer length of 3. The default overlap length is 1.

Sequence encoding is a crucial step in developing topic models. In this analysis, we used a k-mer representation to encode AMPs, similar to n-grams in natural language processing. A k-mer is a contiguous subsequence of length k from an amino acid sequence^32^. To compute k-mers, we chose two main parameters: the extraction method and the length of the k-mers. In our study, we extracted k-mers using overlapped window method to capture detailed information. Shorter k-mers help identify local motifs, while longer ones provide a broader view but can lead to data sparsity. Finding the optimal “k” is important for capturing the biological relevance of AMPs. Thus, we analyzed k-mer lengths from 2 to 20. This range includes short amino acid patterns (2–6 residues) significant for charge or hydrophobicity in antimicrobial activity and longer motifs (10–20 residues) corresponding to structural elements of AMPs. We also developed metrics to assess k-mer length significance in motif analysis. Figure 1 shows an illustration of k-mer generation for a given sequence with length of 12 and a k-mer length of 3.

### Motif extraction: LDA topic model vs frequency-based approaches

LDA is a powerful probabilistic model used to uncover hidden topics within discrete data, such as biological sequences represented as k-mers. As an unsupervised model, it effectively groups co-occurring k-mers that exhibit functional similarities into distinct topics. The topic modeling is a powerful concept, embedding documents as a mixture of topics and defining topics as a mixture of words. In our adaptation of this analysis for motif extraction, we treat sequences as “documents” and k-mers as “words”. Each sequence is viewed as a mixture of latent topics, defined by unique probability distributions^33^. LDA groups k-mers into topics based on a certain probability, and we select the top ten motifs that exhibit high coherence within each topic.

**Fig. 2:**
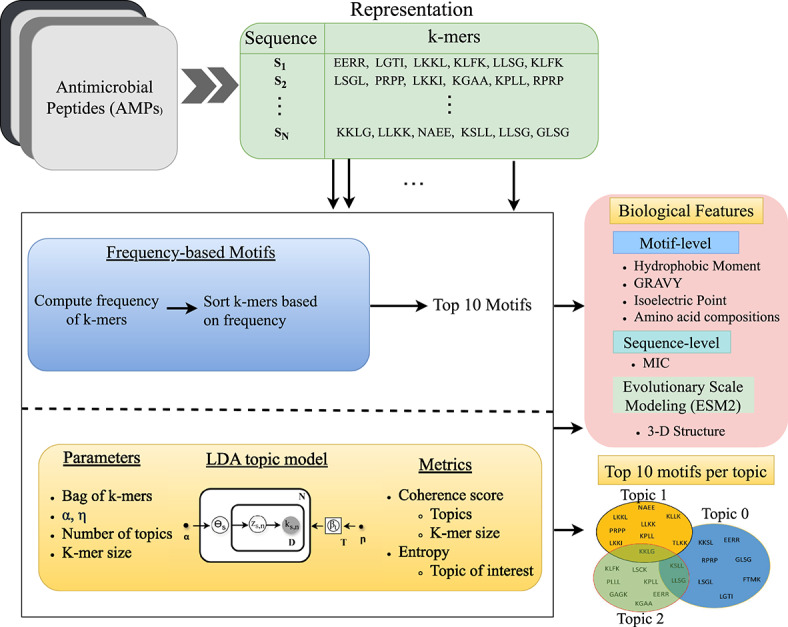
AI-driven motif extraction framework for drug design analysis. This framework extracts k-mers and uses a bag-of-k-mers to represent AMPs. We compute the frequency of k-mers to identify the top 10 motifs from a sequence database. Using these bag-of-k-mers, we train a LDA model, which views each sequence as a mixture of topics, with each topic comprising various motifs, resulting in distinct motif clusters. Key LDA parameters, such as the number of topics and k-mer size, are chosen based on coherence scores, while the topic of interest is identified using an entropy measure. Parameters such as $\alpha$ and $\eta$ regulate the diversity and coherence of motifs in each topic and are set to 0.01 for motif analysis. The model generates clusters of motifs. These clusters are analyzed at both the motif and sequence levels by calculating hydrophobic moment, GRAVY, isoelectric point, amino acid composition, and MIC at the sequence level. Additionally, we utilize evolutionary scale modeling (ESM2) to compute the 3-D structures of these motifs, enhancing our ability to evaluate their biological significance. The analysis also includes motifs extracted through frequency-based method.

In our study, we used Gensim tool^34^ to build the LDA model, applying techniques like variational inference. Training of LDA model, outlined in Algorithm 1, includes bag-of-k-mers similar to the bag-of-words representation in NLP. The initial random assignments of k-mers to topics are refined through iterative reassignment based on topic prevalence and k-mer frequency until convergence is achieved. As shown in Fig. 2, after training, the LDA topic model assigns a probability score to each motif within a topic, indicating how representative that motif is of the topic. By utilizing the co-occurrence of k-mers within the bag-of-k-mers, LDA successfully captures contextual relationships and uncovers hidden patterns in the data. In this study, we selected the top 10 motifs from each topic based on their probability scores to balance interpretability and coverage. The number of motifs considered is a design choice that can be adapted depending on the application. For example, when designing AMPs, a larger number (e.g., top 20 motifs) may be used to enhance sequence diversity, while avoiding motifs with redundant or undesired amino acid patterns such as multiple tryptophan (W) residues.

In topic modeling, the selection of optimal number of topics is crucial, as too few can oversimplify the model, while too many can complicate the analysis. In addition, the number of topics does not directly indicate biological relevance due to the lack of ground truth data. With the true number of motifs unknown, we explored topic models within the 2 to 30 topics range, using the lower bound for minimal separation and the upper bound based on dataset size and motif diversity. We selected the optimal number of topics by maximizing coherence score and conducted additional biological validation at the motif-property level. The selection of LDA hyperparameters ($\alpha$ and $\eta$) is vital for model stability and interpretability while ensuring the biological validity of extracted motifs. In this study, the values of $\alpha$ and $\eta$ are fixed at 0.01, using a fixed seed.

By creating a bag of k-mers, we compute frequency-based motifs. This non-probabilistic approach suggests that the most frequent subsequences represent the motifs effectively. Algorithm 2 illustrates the frequency-based motif extraction process for the AMP analysis. This method allows us to compare motifs generated through LDA models, showing how LDA captures contextual information compared to frequency-based motifs. Such comparisons yield insights into the topic model’s effectiveness in uncovering hidden patterns in the data.

The LDA topic model is an unsupervised learning approach that analyzes the relationships between amino acids in sequences. To understand the relevance of these motifs, we focused on three key physicochemical properties of AMPs: (i) isoelectric point, indicating charge; (ii) hydrophobic character; and (iii) secondary structure. Using the Bio-Python tool^35^, we calculated these properties, which enhanced our understanding of their biological significance. A summary of these properties is available in Section 1 of the Supplementary Material (SI).

### Metrics

We systematically evaluated our AI-driven motif extraction pipeline by leveraging key metrics. Firstly, we utilized the “Coherence Score” to measure the average cosine similarity of motif pairs within each topic, which enabled us to effectively assess and select the most suitable topic model. We also measured entropy to evaluate motif variability, helping us identify the topics of interest and analyze topics by analyzing Minimum Inhibitory Concentration (MIC) values. To enrich our findings, we considered several important properties, including the normalized hydrophobic moment (HM), isoelectric point (IP), GRAVY score, and secondary structure. Additionally, we analyzed the frequency of amino acid residues, quantifying positively charged amino acids that enhance electrostatic interactions with bacterial membranes, as well as hydrophobic residues (L, I, F, V, W) and polar amino acids (S, T, Y) that affect solubility. Finally, we examined the roles of glycine (G) and proline (P) in promoting flexibility in antimicrobial peptide structures^36^. Refer to Sections 2 and 3 in the SI for more information.

**Algorithm 1:**
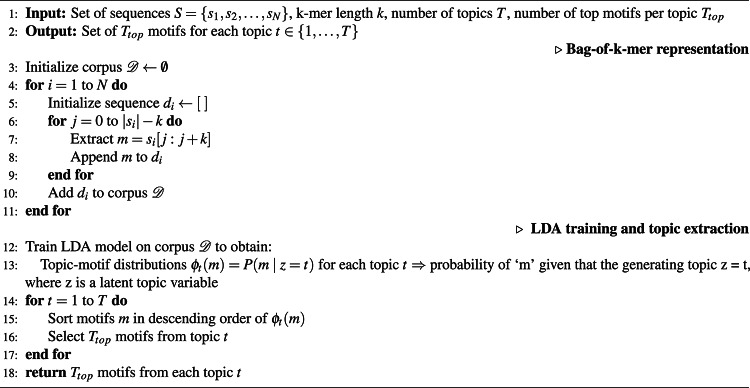
LDA-based motif extraction.

**Algorithm 2:**
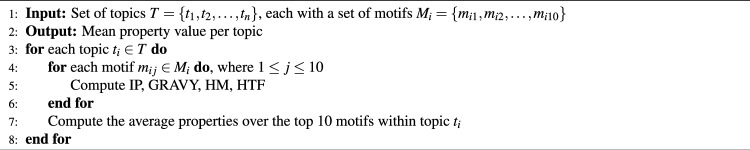
Frequency-based motif extraction.

### Database

To develop topic models that uncover motifs and assess the influence of significant motifs on the antimicrobial activity of peptides, we created a comprehensive dataset focused on linear AMPs composed of standard amino acids. Non-standard amino acids (B, J, O, U, X, and Z) were excluded. We compiled a dataset that includes MIC values standardized to $\upmu$mol/L. For the ranges of MIC values, we calculated the mean, and for values greater than or less than a specific threshold, we used that threshold. When multiple MIC measurements were available for an identical sequences, the mean value is used to account for experimental variability otherwise each sequence is treated as unique. Furthermore, we ensured that any molecular weight unit was accurately converted to $\upmu$mol/L by utilizing the standard molecular masses of the peptides.

**Fig. 3:**
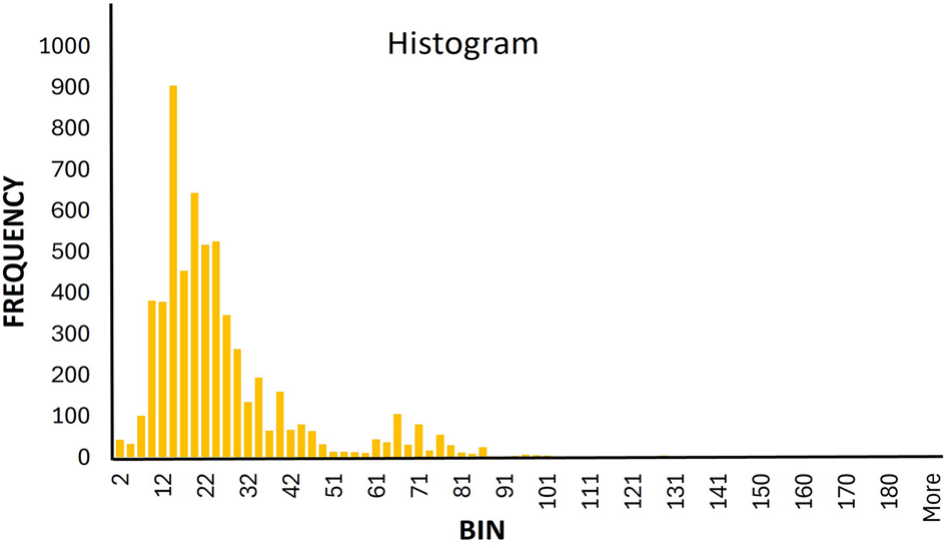
Shows the length distribution of the AMP sequences. As shown, the majority of sequences have lengths between 12 and 32 residues.

Table S1 lists the databases and online sources used for the data collection. The data were initially sourced from three different databases: (i) GRAMPA^37^, which includes information from other databases such as the Antimicrobial Peptide Database (APD)^38^, Database of Antimicrobial Activity and Structure of Peptides (DBAASP)^39^, YADAMP^40^, and DRAMP^41^; (ii) StarPep^42,43^; and (iii) DBAASP3^39^. In this study, we curated a comprehensive dataset of 5, 860 sequences with MIC values against the *E.Coli* target. Figure 3 shows the length distribution of the AMP sequences used for motif analysis.

## Results

### Optimal k-mer size and number of topics

**Fig. 4:**
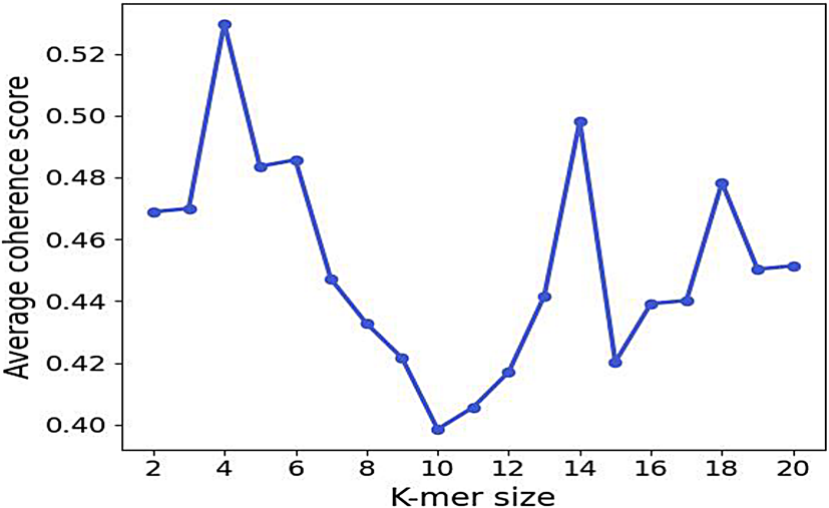
Shows the average coherence score for k-mer lengths from 2 to 20, with topic counts ranging from 2 to 30. We averaged the scores based on 2 to 20 topics for each k-mer length to select the optimal k-mer sizes for motif analysis.

Figure 4 illustrates the average coherence score for k-mer lengths varying from 2 to 20, averaged over topic counts ranging from 2 to 30. In this analysis, we averaged the coherence scores over the topics corresponding to each k-mer length. As illustrated in Fig. 4, we identified peaks at the k-mer lengths of 4, 14, and 18. This suggests that the LDA topic model performs consistently at these specific k-mer sizes, regardless of the number of topics. Notably, the model reliably produced results for motifs likely associated with helical structures, as helices are typically defined by 4 amino acid residues. This is particularly relevant, given that many AMPs are characterized by their helical and amphipathic properties. Moreover, the peak at k-mer lengths 14, and 18 indicates strong reliability for motifs associated with beta sheets.

After selecting k-mer sizes of 4, 14, and 18, we use the coherence score to determine the optimal number of topics for each length. As shown in Fig. S10 in the Supplementary Information, the optimal topics for k-mer lengths of 4, 14, and 18 are 2, 2, and 4, respectively. We further analyze these topics to identify motifs and assess their biological significance in AMP analysis.

**Table 1:** Shows frequency-based and LDA-derived 4-, 14-, and 18-mer motifs.

Method	k-mer size	Topic	Top 10 motifs
LDA-derived	4	0	NAEE, LKKL, KLLK, TLKK, KKLL, PRPP, LLKK, KPLL, KKLG, LKKI
		1	LGTI, KKSL, KSLL, EERR, TLKK, LSGL, FTMK, RPRP, LLSG, GLSG
	14	0	LLSGILGAGKHIVC, LSGILGAGKHIVCG, SGILGAGKHIVCGL
			GLLSGILGAGKHIV, GILGAGKHIVCGLS, ILGAGKHIVCGLSG
			LGAGKHIVCGLSGL, GAGKHIVCGLSGLC, LLIGAGKSAAQSVL
			ALNAAKSAGVSVLN
		1	TGLELMACKITNQC, KTGLELMACKITNQ, GKTGLELMACKITN
			FTLIKGAAKLIGKT, TLIKGAAKLIGKTV, GLFTLIKGAAKLIG
			LFTLIKGAAKLIGK, GKTVAKEAGKTGLE, VAKEAGKTGLELMA
			KTVAKEAGKTGLEL
	18	0	LLSGVLGVGKKIVCGLSG, SGVLGVGKKIVCGLSGLC, GLLSGVLGVGKKIVCGLS
			LSGVLGVGKKIVCGLSGL, ALNAAKSAGVSVLNSLSC,LNAAKSAGVSVLNSLSCK
			SAGVSVLNSLSCKLSKTC,AAKSAGVSVLNSLSCKLS, AKSAGVSVLNSLSCKLSK
			KSAGVSVLNSLSCKLSKT
		1	KTVAKEAGKTGLELMACK, IGKTVAKEAGKTGLELMA, GKTVAKEAGKTGLELMAC
			LIGKTVAKEAGKTGLELM, KEAGKTGLELMACKITNQ, AKEAGKTGLELMACKITN
			TVAKEAGKTGLELMACKI,KGAAKLIGKTVAKEAGKT, FTLIKGAAKLIGKTVAKE
			GAAKLIGKTVAKEAGKTG
		2	LLSGILGAGKHIVCGLSG, GLLSGILGAGKHIVCGLS, LSGILGAGKHIVCGLSGL
			SGILGAGKHIVCGLSGLC, LIGAGKSAAQSVLKTLSC, SAAQSVLKTLSCKLSNDC
			KSAAQSVLKTLSCKLSND,IGAGKSAAQSVLKTLSCK, GAGKSAAQSVLKTLSCKL
			LLIGAGKSAAQSVLKTLS
		3	LKGCWTKSIPPKPCFGKR, ALKGCWTKSIPPKPCFGK, AALKGCWTKSIPPKPCFG
			CVYAYVRVRGVLVRYRRC, IGKEVGMDVIRTGIDVAG, VGMDVIRTGIDVAGCKIK
			EVGMDVIRTGIDVAGCKI,KEVGMDVIRTGIDVAGCK, MDVIRTGIDVAGCKIKGE
			GKEVGMDVIRTGIDVAGC
Freq-based	4	-	FFLG, LFFL, LSLC, LLLL, LLLF, EEER, FLGT, LLFF, LGTI, KSLL
	14	-	MFTLKKSLLLLFFL, FTLKKSLLLLFFLG, LLLFFLGTINLSLC
			LKKSLLLLFFLGTI, TLKKSLLLLFFLGT, LLLLFFLGTINLSL
			KKSLLLLFFLGTIN, KSLLLLFFLGTINL, SLLLLFFLGTINLS
			EEERRDEEVAKMEE
	18	-	KKSLLLLFFLGTINLSLC,ETNAEEERRDEEVAKMEE, DETNAEEERRDEEVAKME
			TNAEEERRDEEVAKMEEI, EEERRDEEVAKMEEIKRG, MFTLKKSLLLLFFLGTIN
			CQDETNAEEERRDEEVAK, QDETNAEEERRDEEVAKM, AEEERRDEEVAKMEEIKR
			NAEEERRDEEVAKMEEIK

The optimal number of topics was determined based on the LDA model’s highest coherence score. Our analysis focuses only on the top 10 motifs identified through LDA and frequency-based methods. Note: “-” means unlike LDA-based model, there are no topic assignments for the frequency-based analysis.

### Motif overlap: LDA-derived vs frequency-based motifs

Table 1 shows motifs derived from LDA and frequency-based methods for 4-mers, 14-mers, and 18-mers. To quantify the motifs extracted using LDA and frequency-based methods, we analyzed the overlap of 4-, 14-, and 18-mer motifs both within LDA and frequency-based methods, as well as across the topics generated by these methods.

Our analysis in Table 2 shows that frequency-based motifs exhibit impressive overlap: all 4-mer motifs are contained within both the 14-mer and 18-mer sets (10/10), and a significant number of 14-mers (7/10) are also found in the 18-mer set, as shown in Figs. S1, S2, and S3 in the SI. In contrast, LDA-derived motifs show less overlap, with only three out of 20 4-mers appearing in the 14-mer and the 18-mer sets. This suggests that LDA captures unique contextual information, as the overlapping motifs are distributed among only five of the 14-mers and eight of the 18-mers. Additionally, while 17 of the 20 LDA 14-mers are present in the 18-mer set, only 14 of the 40 include them (see Figs. S4, S5, and S6). Furthermore, minimal redundancy between LDA-derived and frequency-based motifs enhances their complementary nature, with only two of the LDA 4-mers matching the frequency-based list and no overlap in the 14-mer or 18-mer sets. Overall, these findings highlight that while frequency-based motifs identify prevalent sequences, LDA excels in uncovering unique, context-specific motifs. By leveraging both techniques, we can gain a more comprehensive understanding of the motif structure and sequence functionality in biological datasets, ultimately enriching our insights into biological processes.

**Table 2:** Overlap of motifs within LDA-based and frequency-based motifs of different lengths.

Method	K-mer size	Contained In	Motifs included (s/m)	Motifs containing them (r/k)
Frequency-based	4	14	10/10	10/10
	4	18	10/10	10/10
	14	18	10/10	7/10
LDA model	4	14	3/20	5/20
	4	18	3/20	8/20
	14	18	17/20	14/40

Abbreviation: #Motifs Included: Number of shorter length motifs are contained in motifs of longer length, #Motifs containing Them: Number of Longer length motifs actually contain shorter length motifs . s/m: ‘s’ number of shorter length motifs out of ‘m’ are contained in longer length motifs. r/k: ‘r’ number of longer length motifs out of ‘k’ actually containing shorter length motifs ‘s’. For example, s/m:3/20 indicates that 3 4-mers out of 20 are contained in any of 18-mers; r/k: 5/20: indicates that those 3 of shorter length k-mers appear in 5 out of 20 14-mer motifs.

### Entropy-guided topic selection for motif discovery

In this study, we examined the use of entropy measures to achieve two main goals: 1) identifying topics of interest and 2) distinguishing between diverse and conserved motifs. While the coherence score is useful for assessing topic quality, it has limitations for direct comparisons. Therefore, we implemented entropy measures to clarify motifs from the topic model and highlight areas for further research. Lower entropy values indicate highly conserved motifs that are likely stable and significant evolutionarily, though not all conserved motifs have distinct biological functions. In contrast, higher entropy values suggest increased variability or noise, while motifs with intermediate values often relate to known AMP domains. In Table S2 of the SI, we present detailed entropy values for motifs from frequency-based methods alongside those from the LDA model. Frequency-based motifs exhibited lower entropy (below 2.5) for both 4-mer and 14-mer motifs, indicating a tendency towards repetitive patterns that may limit functional diversity. Conversely, the LDA model revealed varying mean entropy values across 4-mer, 14-mer, and 18-mer motifs, highlighting significant diversity and complexity in the identified motifs. Notably, the entropy of 18-mer motifs from the LDA model in topic 3 was higher than that of frequency-based motifs, indicating a richer exploration of sequence variability and potential functional diversity. Although this trend was less consistent for 4-mer motifs in topic 0, it suggests promising areas for future research.

### Motif analysis

As mentioned in Section , we identified optimal k-mer sizes of 4, 14, and 18 based on LDA model coherence scores, resulting in 2 topics for k-mer sizes 4 and 14, and 4 topics for k-mer size 18. To explore the biological significance of the motifs related to antimicrobial, we used two approaches: 1) calculating motif-level properties and 2) mapping sequence-level attributes to motifs.

-   **Motif-level property comparison:** In this approach, we assess motif properties across topics to uncover their biological significance. The algorithm for quantifying motif relevance, based on HM, GRAVY, IP, Helix and Turn Fraction (HTF), is outlined in Algorithm 3.
-   **MIC sequence-level comparison:** Sequence-level properties were mapped using motif-level aggregation and topic-level summarization. This approach helps in understanding how different motifs influence MIC values across topics. The procedure for quantifying motif relevance per topic through MIC association is described in Algorithm 4.

**Algorithm 3:**
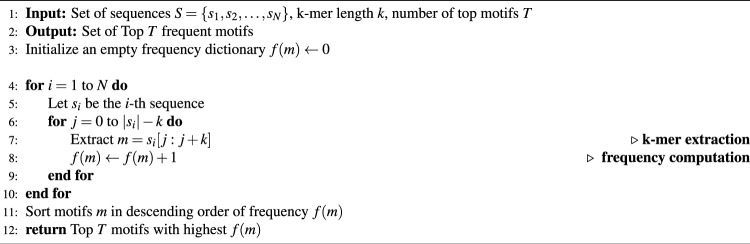
Motif-level property comparison: quantifying motif relevance per topic using HM, GRAVY, IP, HTF properties computed at motif level.

**Algorithm 4:**
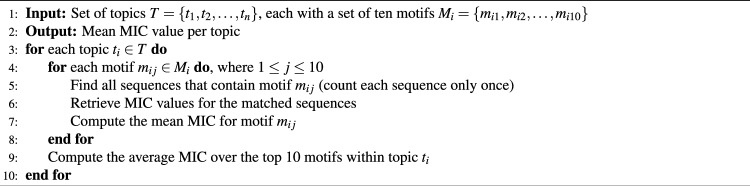
MIC sequence-level comparison: quantifying motif relevance per topic by MIC association at sequence level.

### Comparison 1: motif-level properties

We calculated three different properties at the motif level: isoelectric point, hydrophobic moment, and GRAVY. These properties were used to analyze the motifs extracted using the LDA-based and frequency-based methods. Additionally, we generated 3D structures for longer motifs (14-mer and 18-mer) using ESM Fold software. To enhance our understanding of the amino acid compositions of the motifs derived from both methods, we determined the frequency of the top motifs and visually assessed the differences and significance of the motifs extracted using each approach.

**Fig. 5:**
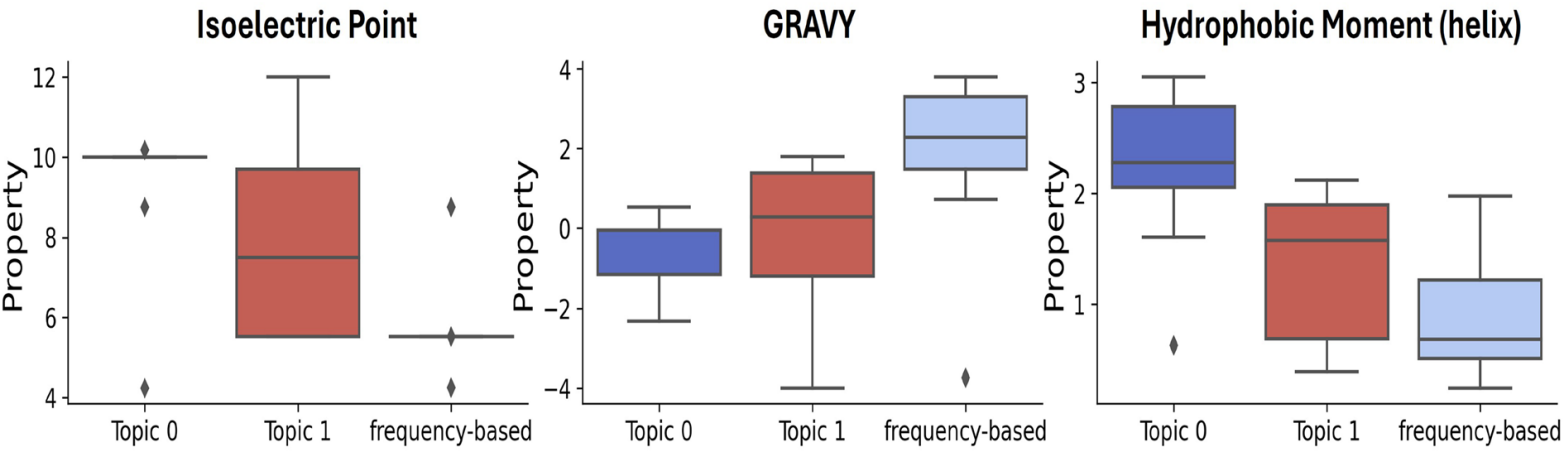
The distribution of motif properties for the top 10 4-mer: LDA model-derived motifs and frequency-based motifs. Motifs in topic 0 show a higher IP than those in topic 1, indicating that topic 0 motifs are more basic, while frequency-based motifs tend to be more acidic. In terms of hydrophobic moment, topic 0 motifs show higher values as compared to topic 1 motifs, whereas frequency-based motifs display lower values than both topics. This suggests that topic modeling-derived motifs are more amphipathic. Additionally, the lower GRAVY index of both topic 0 and topic 1 motifs compared to frequency-based ones opens new avenues for exploring their interactions in biological contexts.

**4-mer motifs:** From Fig. 5, it is evident that the motifs in Topic 0 display a higher IP than those in Topic 1, which have a broader range typically spanning from 5 to 12. Conversely, motifs based on frequency analysis exhibited lower IP values than those derived from topics 0 and 1. It is interesting to note that motifs generated through LDA are basic, whereas frequency-based motifs are on the more neutral side. Additionally, the motifs in Topic 0 had a greater HM than those in Topic 1. In contrast, frequency-based motifs demonstrated a lower HM than both Topics 0 and 1, indicating that motifs derived from the topic modeling approach are more amphipathic than frequency-based motifs. Furthermore, both Topic 0 and Topic 1 motifs exhibited a lower GRAVY index than frequency-based motifs.

**Fig. 6:**
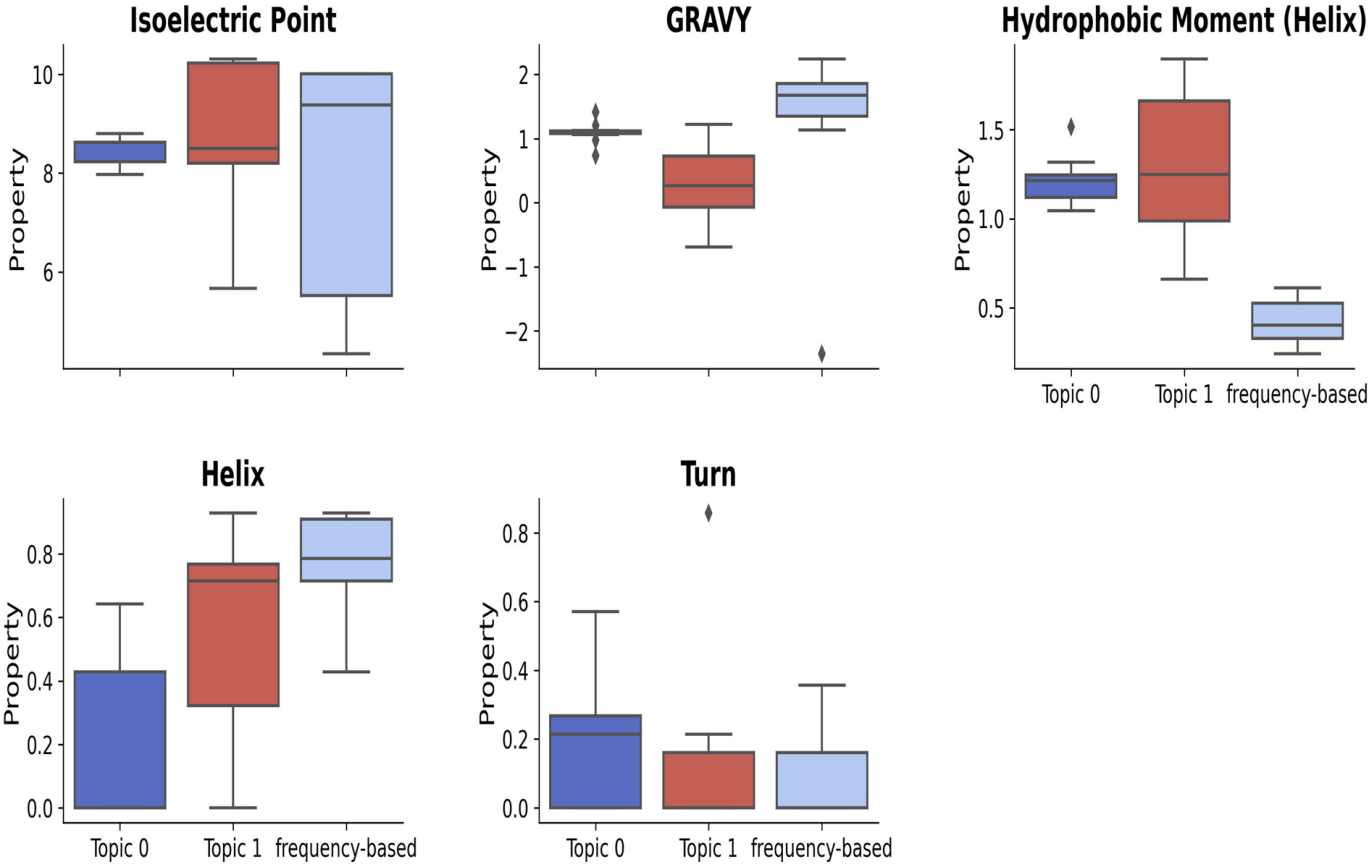
An overview of motif-level feature distributions for the top 10 14-mer LDA- and frequency-based motifs related to *E. Coli.* The motifs in topics 0 and 1 have IP values above 7, indicating they are predominantly basic, while frequency-based motifs show a broader spectrum, including both basic and acidic. Frequency-based motifs also have a higher GRAVY index, indicating greater hydrophobicity, with topic 0 motifs showing a particularly high index. Both topics 0 and 1 exhibit higher hydrophobic moments, but topic 0 has less variability. In contrast, frequency-based motifs have a lower hydrophobic moment. Regarding helix and turn fractions, topics 0 and 1 present unique helix fraction ranges, while frequency-based motifs tend to have higher helix fractions. Both motif types exhibit similar trends in turn fractions, highlighting the complementary insights provided by topic modeling and frequency analysis in understanding motif characteristics.

**14-mer motifs:** Figure 6 shows the motif-level feature distributions for the top 10 14-mer LDA- and frequency-based motifs. Motifs in topics 0 and 1 have IP values above 7, indicating they are primarily basic, while frequency-based motifs show a wider range, reflecting both basic and acidic characteristics. Frequency-based motifs also exhibit a higher GRAVY index than those from topic models, with topic 0 having a notably higher index than topic 1. Both topics demonstrate elevated hydrophobic moments, though topic 0 is less variable. In contrast, frequency-based motifs have lower hydrophobic moments, suggesting that topic model motifs are more amphipathic. Regarding helix and turn fractions, topics 0 and 1 show distinct ranges of helix fractions, while frequency-based motifs generally have higher helix fractions. Nonetheless, both types maintain similar trends in turn fractions, highlighting valuable insights for future research.

**Fig. 7:**
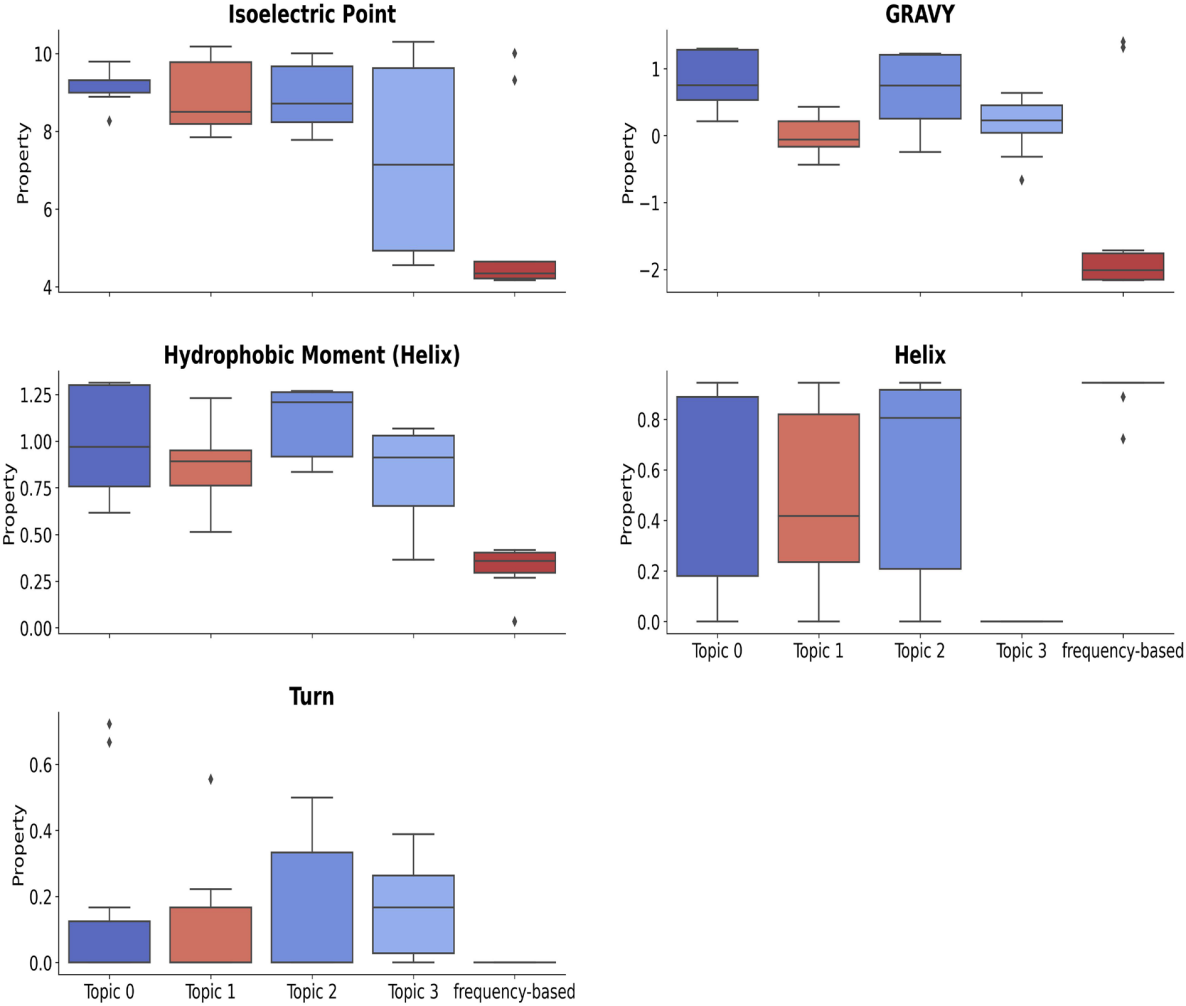
The distributions of motif-level properties of LDA-derived and frequency-based 18-mer motifs. The LDA-derived motifs found in topics 0-3 exhibit higher values for IP, GRAVY, and HM compared to the frequency-based motifs. This suggests that the LDA-derived motifs are hydrophobic, amphipathic, and basic. In contrast, frequency-based motifs show lower values for hydrophobicity, IP, HTF. Notably, topic 3 motifs display lower values for helix fraction, hydrophobicity, and long-range isoelectric point.

**18-mer motifs:** Figure 7 shows that LDA-derived motifs in topics 0-3 have higher values for IP, GRAVY, and HM compared to frequency-based motifs, indicating they are hydrophobic, amphipathic, and basic. In contrast, frequency-based motifs exhibit lower values for hydrophobicity, IP, and helix/turn fractions. Notably, topic 3 motifs display lower helix fraction and hydrophobicity, suggesting they can be both acidic and basic.

In summary, frequency-based motif analysis reveals how often motifs appear across sequences, providing a general overview of patterns. However, this method can be repetitive and may lack biological relevance due to the absence of context. The LDA topic model, on the other hand, captures semantic context and uncovers hidden structures, effectively grouping motifs with similar properties in a biological framework.

### Comparison 2: amino acid compositions

**Fig. 8:**
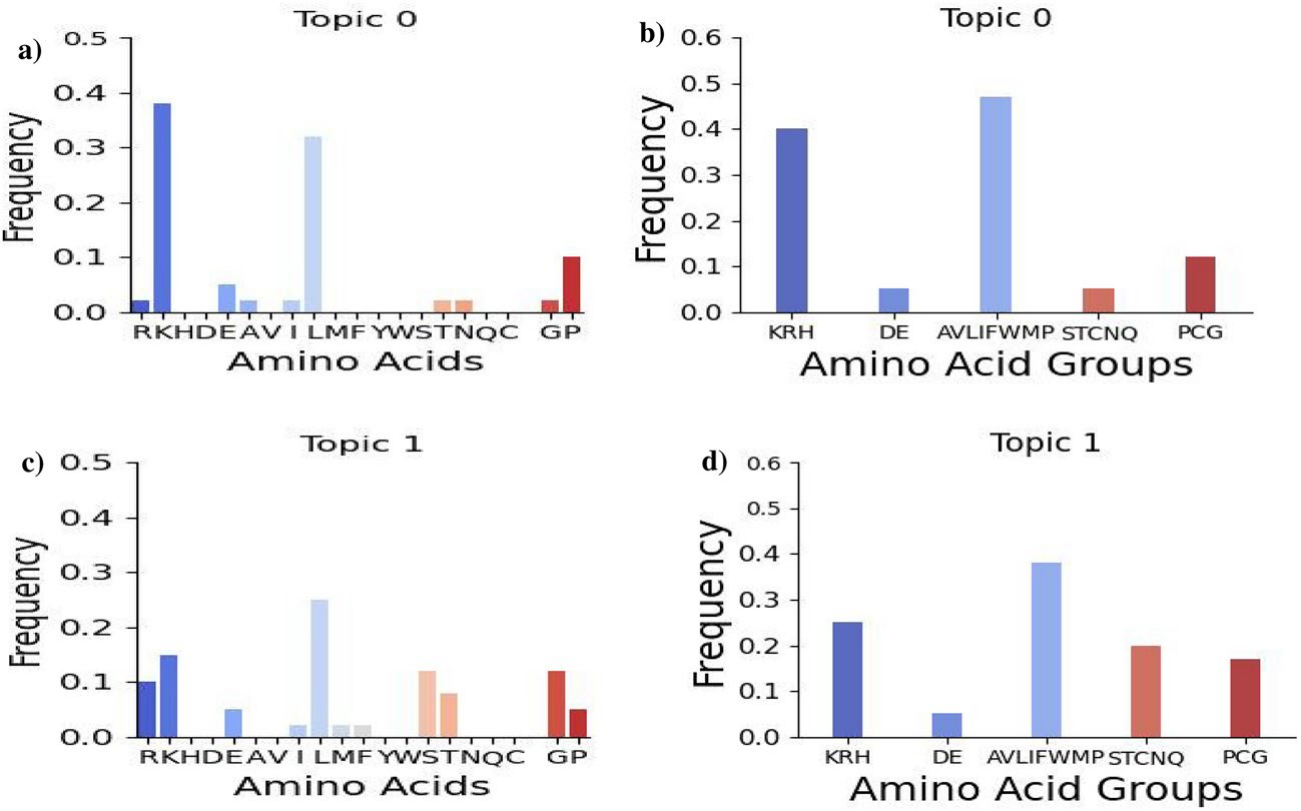
Amino acid compositions for the top ten LDA-derived 4-mer motifs associated with the *E. coli* target. It depicts both individual amino acid compositions (a and c) for each of the top motifs and group-wise compositions (b and d) of topic 0 and 1. Specifically, it includes the amino acid compositions for positively charged amino acids (K, R, H), negatively charged amino acids (D, E), hydrophobic amino acids (A, V, L, I, M, F, Y, W), polar uncharged amino acids (S, T, N, Q), and special cases of amino acids (C, G, P).

**Fig. 9:**
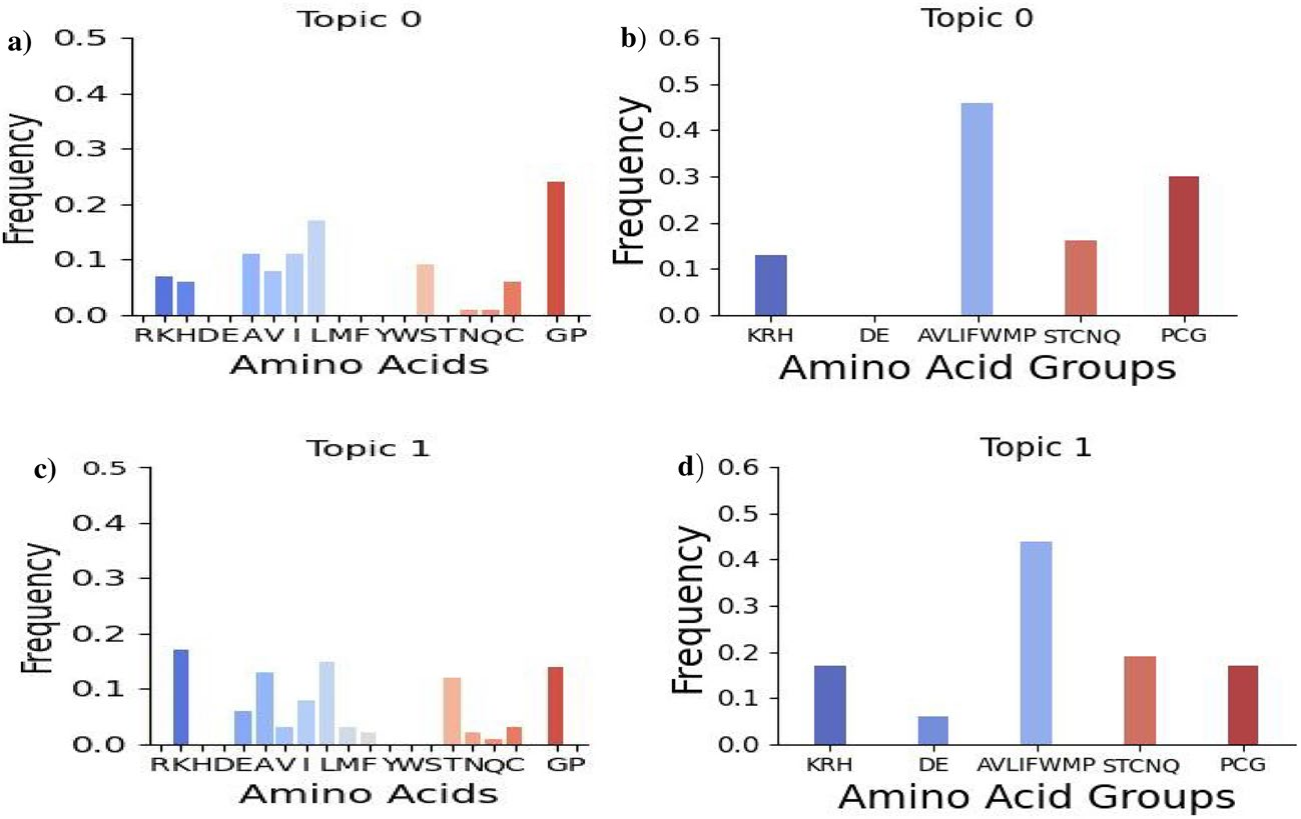
Amino acid compositions for the top ten LDA-derived 14-mer motifs associated with the *E. coli* target. It depicts both individual amino acid compositions (panels a & c) for each of the top motifs and group-wise compositions (panels c and d) of topic 0 and 1.

Table 1 outlines the LDA-derived and frequency-based motifs for 4-mers, 14-mers, and 18-mers. To enhance our understanding of the amino acid compositions within these motifs, Figs. 8,  9, S7, and S8 (found in the SI) present both individual and grouped amino acid compositions. The grouped compositions categorize amino acids as positively charged (K, R, H), negatively charged (D, E), hydrophobic (A, V, L, I, M, F, Y, W), polar uncharged (S, T, N, Q), and special cases (C, G, P). This concise analysis offers valuable insights into the motifs identified through the LDA topic model and frequency-based methods.

**4-mer motifs**: The analysis in Fig.  8 highlights the notable concentration of proline residues in LDA model-derived 4-mer motifs. These residues help maintain extended, open structures in peptides, facilitating their interaction with bacterial membranes. Proline-rich motifs, like ”RPRP,” often feature positively charged amino acids such as arginine, enabling effective engagement with negatively charged lipids in these membranes^44,45^. This capability allows proline-rich AMPs to penetrate bacterial membranes without immediate cell lysis, reducing cytotoxicity while preserving antibacterial activity. Additionally, the motif compositions reveal a balance between charged and uncharged residues, which is critical for the hydrophobic properties of AMPs. Notably, Topic 1 (c & d) has a lower fraction of positively charged residues and a higher fraction of polar uncharged residues compared to Topic 0 (a & b).

**14-mer motifs:** Figure 9 shows the amino acid compositions of the top 10 14-mer motifs. Topic 1 (panels c & d) has a higher composition of positively charged residues but fewer special case residues, suggesting intriguing functional implications. In contrast, topic 0 (panels a & b) contains more glycine-rich motifs. Both topics display significant presence of cysteine and histidine residues, with cysteine being essential for disulfide bridge formation that stabilizes the beta-strand structure of AMPs^46–48^. Topic 0’s high concentration of histidine residues provides pH-responsive cationic properties and facilitates metal binding, promoting membrane disruption and reactive oxygen species generation at acidic sites^49,50^. Its structural roles enhance antimicrobial efficacy while minimizing host cell toxicity^51–53^.

**18-mer motifs:** Figure S7 presents an analysis of the amino acid compositions (Panels a, c, e, & g) and the group-wise aggregated compositions (b, d, f, & h) of 18-mer motifs across four topics. Topics 1 and 3 show enrichment in positively charged residues like lysine, which enhances antimicrobial activity through stronger interactions with negatively charged bacterial membranes. Conversely, Topics 0 and 2 are rich in uncharged residues, particularly serine (S), suggesting flexibility or potential phosphorylation sites. Glycine (G) is prevalent in all topics, highlighting its role in structural flexibility. In addition, hydrophobic residues are dominant across all topics, essential for membrane interactions. Notably, Topic 1 has a unique enrichment of threonine (T), which could influence motif polarity or function through hydrogen bonding. These variations indicate that motifs associated with each topic may serve distinct structural or functional roles, reflecting the underlying biological mechanisms relevant to AMP design.

**Frequency-based motifs:** Figure S8 shows the amino acid compositions for 4-, 14-, and 18-mer motifs from a frequency-based method (Panels a, c, & e) along with their group-wise compositions (b, d, & f). Shorter motifs (4- and 14-mers) are enriched in leucine and phenylalanine, leading to higher hydrophobicity. This characteristic aids in anchoring peptides in the bilayer through hydrophobic interactions, potentially causing membrane thinning and the formation of defects or pores^54,55^. In contrast, the 18-mer motifs exhibit greater diversity, with an increase in both positively and negatively charged residues. This balanced charge distribution may enhance functional complexity, supporting interactions such as forming amphipathic helices or engaging in electrostatic interactions with microbial membranes.

In summary, frequency-based motifs are rich in leucine and phenylalanine, especially in the 4- and 14-mer lengths, contributing to their strong hydrophobic properties. In contrast, LDA-derived motifs show lower phenylalanine levels but maintain hydrophobicity through alternative residues and exhibit increased lysine presence. This highlights the robust hydrophobic characteristics of frequency-based motifs while suggesting that the compositional diversity in LDA-derived motifs may lead to distinct antimicrobial functions.

### Comparison 3: structural analysis

**Fig. 10:**
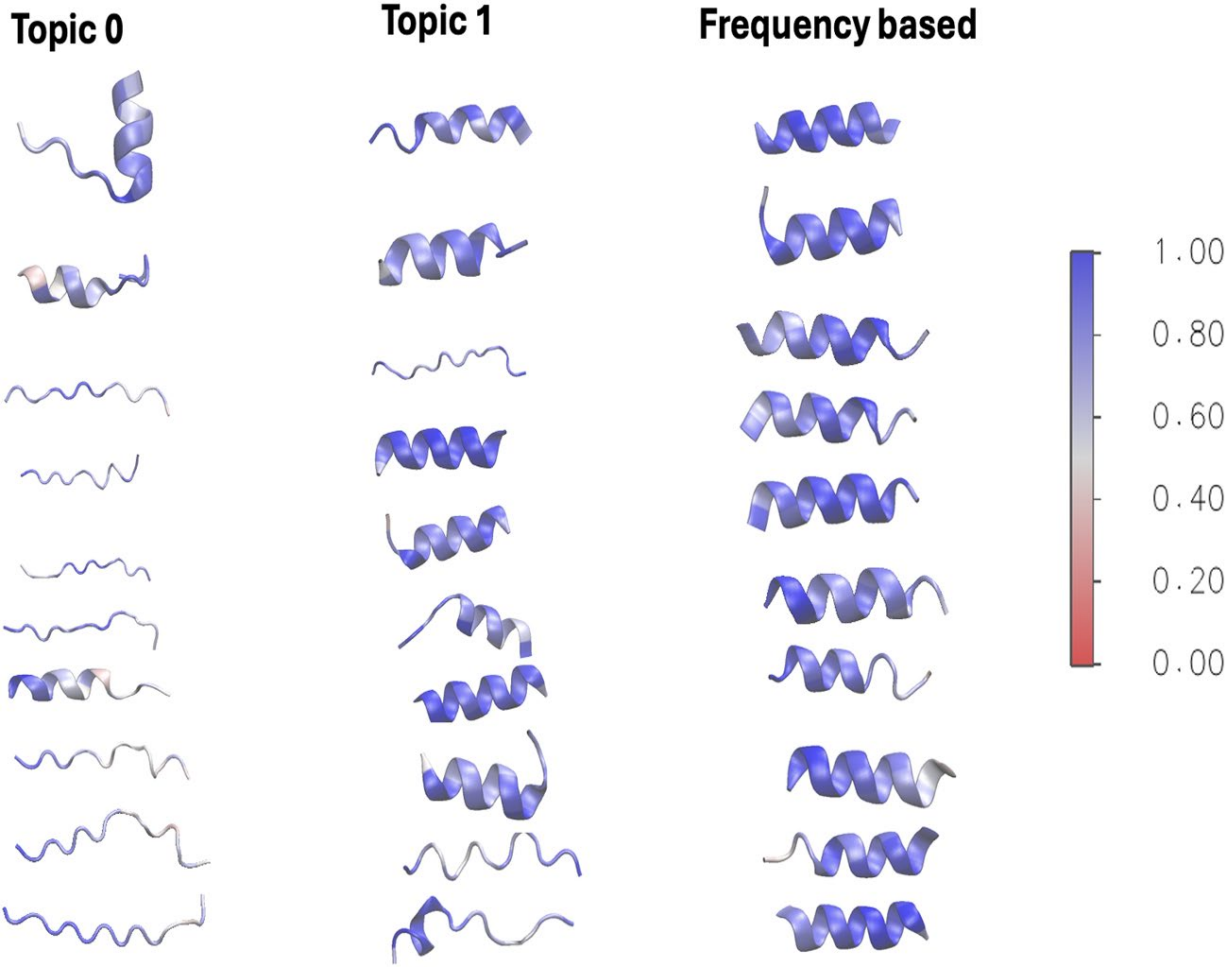
The structural analysis of the top ten LDA- and frequency-based 14-mer motifs. Topic 0 primarily exhibit random coil structures, with the last three motifs displaying helices, while Topic 1 showcases helical structures, except for the 8th and 9th motifs. Frequency-based motifs show a more uniform structure overall, indicating they are less common than LDA-derived motifs in AMP analysis. Additionally, the color-bar indicates the confidence levels of the predicted structures as determined by the ESMFold model.

To better understand the motifs of the LDA topic model and frequency-based approaches, we analyzed their structural characteristics using the ESM fold model^17^. As shown in Fig.  10, the structural quality of 14-mers motifs was visualized using the pLDDT color map of ESMFold. Although ESMFold is not specifically optimized for short peptides, predicted models were used to qualitatively illustrate structural diversity between topics, with confidence levels indicated by the pLDDT scores. The motifs of topic 0 mainly exhibited random coil structures, while the last three showed some helical properties. In contrast, topic 1 motifs predominantly displayed helical structures, but the eighth and ninth motifs were exceptions, suggesting opportunities for further research into their structural capabilities.

**Fig. 11:**
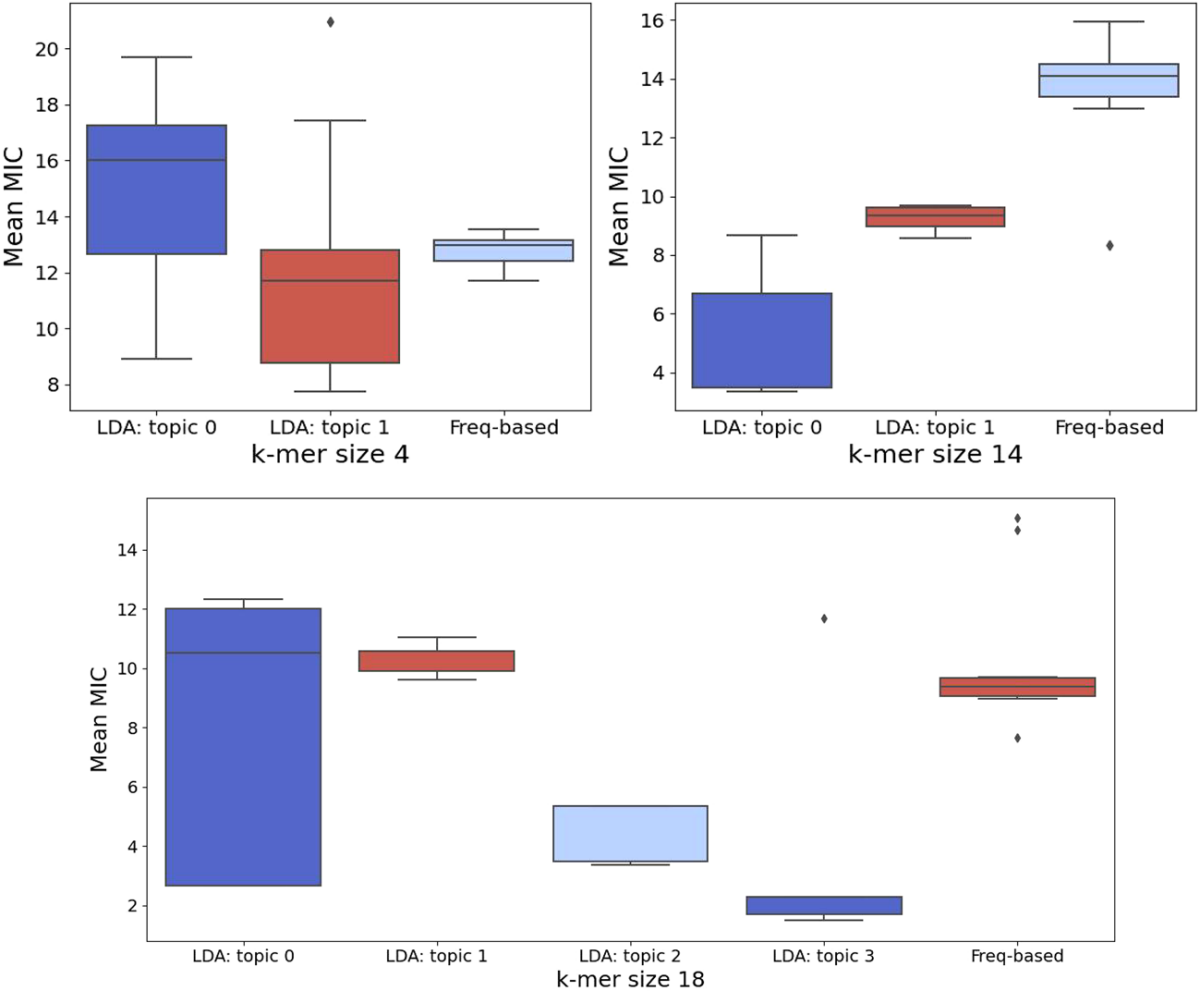
Mean MIC values for LDA- and frequency-based motifs of lengths 4, 14, and 18. For 4-mers, LDA-derived motifs in topic 1 show lower MIC values than those in topic 0. In 14-mers, topics 0 and 1 have lower MIC values compared to frequency-based motifs. for 18-mer motifs, Topics 2 and 3 exhibit the lowest MIC values relative to topics 0 and 1, while frequency-based motifs consistently show higher MIC values, especially in topic 1.

Additionally, Fig.  11 indicates that topics 0 and 1 had lower MIC values for 14-mer sequences, highlighting Topic 1’s potential as candidate AMPs. However, Frequency-based motifs demonstrated a more uniform structure. Figure S9 in the SI presents the structural analysis of the top ten LDA- and frequency-based 18-mer motifs. Here, topics 0-2 primarily exhibited helical structures, with only three showing random coils, while Topic 3 consisted entirely of random coils. This suggests distinct structural properties, and frequency-based motifs showed consistent structural uniformity, similar to the 14-mer motifs.

## COMPARISON 4: sequence-level property (MIC)

This section compares LDA-derived motifs with frequency-based motifs through MIC sequence-level analysis. We assessed motif significance by examining their occurrences in actual sequences and correlating them with MIC values. This provides an approximation of motif potency but may not fully reflect the true activity of motifs in isolation. To complement this, in previous section we computed chemical properties at the motif level, enabling more reliable comparisons and interpretation of motif activity.

For instance, Fig. 11 shows that for 4-mer motifs, LDA-derived motifs in Topic 1 had lower MIC values than those in Topic 0, while frequency-based motifs also showed lower MIC values than Topic 0. For 14-mer motifs, frequency-based motifs exhibited higher MIC values than LDA-derived ones, with Topic 0 having the lowest values. In terms of 18-mer motifs, Topics 2 and 3 demonstrated the lowest MIC values, whereas frequency-based motifs correlated with higher MIC values in Topic 1. Overall, these findings suggest that motifs derived from the LDA model are more closely associated with sequences displaying lower MIC values.

## Discussions

When using multiple databases, we have taken into account about redundant sequences by taking the average MIC value. However, highly similar sequences can lead to over representation of specific k-mers. Although frequency-based analyzes are affected by this redundancy, topic modeling uncovers hidden structures beyond high-frequency motifs. Our comparison of an LDA-based approach with a frequency-based method demonstrated that LDA effectively identifies biologically significant motifs, even in the presence of overlapping sequences across databases.

We evaluated the topic model by extracting shorter and longer motifs. Shorter motifs are particularly useful for generating diverse AMPs, while longer motifs capture richer contextual information within peptide sequences. For novel sequence design, we prioritize shorter motifs (e.g., 4-mers) to promote diversity, whereas longer motifs can be employed to design AMPs that are more similar to known targets. Thus, motif length serves as a design parameter that can be tuned depending on the desired balance between diversity and similarity when exploring new AMP candidates.

Frequency-based motif discovery demonstrates comparable MIC values at shorter motif lengths (4-mers), but LDA-derived motifs show significant advantages at longer lengths (14- and 18-mer motifs) in MIC association and diversity. This indicates that frequency alone may overlook essential long-range dependencies and contextual relevance crucial for understanding biological functions. Our findings highlight the effectiveness of topic modeling in revealing latent motif structures that carry functional insights. The LDA-derived motifs of topics 0 and 1 have MIC profiles similar to frequency-derived motifs at k-mer = 4, but topics 0 and 1 present significantly lower MIC values (p < 0.001 in Table 3) for 14-mers, suggesting contextually relevant motifs missed by frequency-based methods.

Similarly, LDA-derived motifs of topics 2 and 3 yield lower MIC values for 18-mers. Mapping sequence MIC values to motifs and aggregating them based on occurrences can uncover connections between specific motifs and varying MIC levels, offering insights into AR. However, overlapping box plots may emerge when few sequences show outlier MIC values, complicating clear topic separations. The biological complexity also plays a role, as multiple factors influence the MIC, indicating that motifs alone may not fully account for these variations. Some biophysical properties of analyzed peptide motifs may not be entirely accurate, and our assumption of helical formation when calculating hydrophobic moments needs further validation.

Comparison of LDA motifs with those of other methods is challenging. LDA motifs are fixed-length sequences (e.g., 4, 14, or 18-mers), while deep learning^56^ and attention-based models^57^ produce longer variable-length motifs. These models prioritize predictive accuracy over recurring sequences, whereas LDA identifies co-occurring fragments without supervision. The different methodologies make meaningful comparisons complex, but they are worth exploring in future research.

**Table 3:** Shows the mean MIC for frequency-based and LDA-derived motifs computed using MIC sequence-level comparison, revealing that 14-mers from topics 0 and 1 had significantly lower MIC values (equal-variance t-test with p_value < 0.001).

K-mer size	Frequency-based (mean MIC)	Topic	LDA model (mean MIC)	p_value
4	12.73 ± 0.20	0	15.09 ± 1.13	0.95
		1	12.08 ± 1.34	0.05
14	13.61 ± 0.64	0	5.76 ± 0.67	< 0.001
		1	9.28 ± 0.13	< 0.001
18	10.26 ± 0.78	0	7.97 ± 1.46	0.45
		1	10.33 ± 0.16	0.98
		2	4.58 ± 0.31	< 0.001
		3.	2.98 ± 0.97	< 0.001

This suggests a high probability of rejecting the null hypothesis that the mean MIC of LDA-derived motifs is greater than that of frequency-based motifs.

## Conclusion and future work

We effectively demonstrated the LDA topic model’s ability to uncover motifs linked to antimicrobial activity. Our method involves segmenting AMPs into k-mers, building an LDA model, and mapping motifs to relevant biological properties. Our results reveal that arginine (R)-rich sequences are particularly effective AMPs, while lysine (K) and leucine (L) motifs also show significant antimicrobial activity with lower MIC values. The LDA model successfully extracts contextually relevant motifs, which show lower MIC values as compared to frequency-based method. In conclusion, motifs are essential for identifying promising candidates for AMP design. By comparing the motifs present in low and high MIC groups, we see a clear correlation between specific motifs and antimicrobial effectiveness. These characteristics can be combined with various properties to enhance prediction and classification tasks, advancing our understanding of AMPs. In future work, we aim to utilize the identified motifs to design novel AMPs and evaluate their biochemical and biological properties, further exploring the significance of these motifs and the patterns revealed by our analysis.

## Supplementary Information

Supplementary Information.

## Data Availability

The code utilized for our experiments can be accessed at https://gitlab.com/padi.sarala-group/Topic_Model_Motif_Discovery. The folder contains scripts for topic model optimization, metrics, data analytics for biological outputs, pretrained weights for structure prediction using ESM fold, and feature extraction using Bio-Python.
