# Supplementary material for: AI-driven antimicrobial peptide characterization unveils novel motifs for drug design
Source: Sci Rep. 2025 Dec 29;16:829. doi: 10.1038/s41598-025-30419-1 (PMC12780253; doi:10.1038/s41598-025-30419-1)
Supplement: Supplementary file 1 — Supplementary Information. [file 41598_2025_30419_MOESM1_ESM.pdf]

Supplementary Material

Sarala Padi[1], Kinjal Mondal[1, 3, 5], David Hoogerheide[2], Frank Heinrich[2,4], Mihaela Mihailescu[3], Jeffery B. Klauda[5,6], and Antonio Cardone[1]

[1]Information Technology Laboratory (ITL), NIST, Gaithersburg, USA
[2]NIST Center for Neutron Research (NCNR), NIST, Gaithersburg, USA
[3]Institute for Bioscience and Biotechnology Research (IBBR), Rockville, USA
[4]Department of Physics, Carnegie Mellon University, Pittsburg, PA, USA.
[5]Institute for Physical Science and Technology and Biophysics Program, University of Maryland, College Park, Maryland, USA
[6]Department of Chemical and Biomolecular Engineering, University of Maryland, College Park, Maryland, USA

November 21, 2025

1 AMP-relevant biochemical properties

- **Isoelectric Point [1]:** Isoelectric point (IP) is the pH at which a peptide motif has no net electrical charge. This concept is crucial for understanding the behavior of peptides. Higher IP values indicate a greater concentration of basic amino acids, such as arginine and lysine, whereas lower IP values suggest a higher proportion of acidic residues, such as aspartic acid. Generally, motifs with an IP below 7 are acidic, and those above 7 are basic. This classification helps to predict the overall charge of peptides, particularly antimicrobial peptides (AMPs), which are often cationic. Understanding the isoelectric point enhances our knowledge ofthe peptide structure and function.

- **Hydrophobic Character**: To quantify the hydrophobic character of the peptides, we used two different quantities (i) GRAVY (Grand Average of Hydropathy) [2], and (ii) hydrophobic moment [3]. The GRAVY value is the sum of the hydropathy/hydrophobicity values of the amino acids in a sequence divided by the total number of amino acids. Overall, this indicted the hydrophobicity of the peptide segment in general. The hydrophobic moment provides an overall measure of peptide amphiphaticity. It is computed as:

$$\mu_{H_m} = \frac{1}{n}\sqrt{\left(\sum_{i=1}^{n} H_{m_i}\cos\theta_i\right)^2 + \left(\sum_{i=1}^{n} H_{m_i}\sin\theta_i\right)^2}$$
$$\theta_i = (i-1)\Delta, \quad \Delta = 100^\circ \ (\alpha\text{-helix}) \tag{1}$$

where 'H_{m_i}' denotes the hydrophobicity of the ith amino acid, $\hat{\theta}_i$ denotes the unit vector from the direction of the nucleus of the α carbon to the geometric center of the side chain of the ith amino acid. Δ denotes the angle at which the successive side chains emerge from the backbone. This quantity is more related to the $3D$ structure of the peptide and amphiphaticity than GRAVY. We calculated the hydrophobic moment, assuming that the entire motif was a helix.

- **Secondary structure propensity:** This is a rough estimate of the approximate helix and turn fractions present in the peptide predicted from the ESMFold [4] model. When antimicrobial peptides

interact with the membrane, some of them form helices, beta-sheets, and coils [5]. It should be noted that these predicted structures might be more biased towards the structures assumed by these peptides in the solution rather than in the membrane as the training data for ESMFold are mostly peptide structures in the solution. This will give us the idea of the secondary structure propensity for AMPs.

- **Minimum Inhibitory Concentration (MIC):** MIC is a crucial metric for assessing bacterial susceptibility to antibiotics through in vitro tests [6]. It represents the lowest concentration of an antimicrobial agent, such as AMP, which inhibits microbial growth after incubation and is measured in µg/mL or µM. Lower MIC values indicate higher potency.

2 Coherence:

Evaluating LDA topic models is important to understand the quality and relevance of topics in a specific domain. The coherence score measures the degree of semantic similarity between the top N [1] motifs in each topic, with higher coherence scores indicating better topic quality [7]. There are multiple methods for computing the coherence measure. We calculate the coherence score by combining point-wise mutual information (PMI) with cosine similarity [8], which is defined as:

Topic Coherence (C_v) is the average of the cosine similarities over all motif pairs (m_i, m_j) in topic:

$$C_v = \frac{1}{|T| \cdot (|T| - 1)} \sum_{i=1}^{|T|} \sum_{j=i+1}^{|T|} \text{Similarity}(m_i, m_j) \tag{2}$$

Where "T" is the number of topics, and $|T|$ is the number of motifs in the topic. The coherence between the two motifs m_i and m_j in a topic is calculated using the cosine similarity of the context vectors:

$$\text{Similarity}(m_i, m_j) = \frac{\mathbf{v}(m_i) \cdot \mathbf{v}(m_j)}{\|\mathbf{v}(m_i)\| \|\mathbf{v}(m_j)\|} \tag{3}$$

where a context vector $v(m)$ is built for each motif "m'' in a topic, where the vector components represent co-occurrence of "m'' with other motifs in a predefined sliding window over the corpus of k-mers.

$$v(m) = [\text{NPMI}(m, m_1), \text{NPMI}(m, m_2), \ldots, \text{NPMI}(m, m_n)] \tag{4}$$

Where "m'' is the target motif and $m_1, m_2, \ldots, m_n$ are other motifs in the same topic, $NPMI(m, m')$ is the normalized pointwise mutual information between m and m'.

Normalized Pointwise Mutual Information (NPMI) measures the association between two motifs based on their co-occurrence probabilities and is given by:

$$\text{NPMI}(m, m') = \frac{\log \frac{P(m,m')}{P(m)P(m')}}{-\log P(m, m')} \tag{5}$$

Where P(m,m') is the joint probability of motifs m and m' occurring together, and $P(m)$ and $P(m')$ are the individual probabilities of m and m'.

3 Entropy:

Entropy provides a measure of the uncertainty of the random variables. It is sometimes called missing information: the larger the entropy, the less *a priori* information one has on the value of the random variable [9, 10]. At each position 'i', the entropy of an amino acid residue in a motif can be described as:

[1] For this study, we only examined the top 10 motifs in each topic.

$$H_i = -\sum_{a=1}^{21} f_{a,i} \times log_2(f_{a,i}) \tag{6}$$

where $f_{a,i}$ is the relative frequency of amino acid 'a' at position 'i'. The maximum possible entropy for amino acids is $-log_2(21) \approx 4.39$ bits, assuming complete randomness across the 21 amino acids.

Table S1: shows a list of the databases and websites used to obtain data for motif analysis.

**Database Name**	**Online Source**
GRAMPA	https://github.com/zswitten/Antimicrobial-Peptides/blob/master/data/grampa.csv
APD	https://aps.unmc.edu/
DBAASP	https://dbaasp.org/home
YADAMP	https://webs.iiitd.edu.in/raghava/satpdb/catalogs/yadamp/
DRAMP	http://dramp.cpu-bioinfor.org/
StarPep	https://github.com/Grupo-Medicina-Molecular-y-Traslacional/StarPep
DBAASP3	https://dbaasp.org/home

Table S2: Presents entropy values of frequency- and LDA-derived motifs. Frequency-based motifs consistently show lower entropy (below 2.5) for 4-mer and 14-mer motifs, indicating repetitive patterns that limit functional diversity. In contrast, LDA-extracted motifs exhibit higher mean entropy values above 2.5 for 4-mer, 14-mer, and 18-mer motifs, with topic 3 showing higher values for 18-mer, suggesting these motifs are more diverse and functionally relevant, warranting further investigation.

Method	**k-mer size**	**Topic**	**Entropy(μ)**	**Std(σ)**
Frequency-based	4	-	2.075	0.385
	14	-	2.366	0.367
	18	-	2.654	0.311
LDA-derived	4	0	2.04	0.22
		1	2.59	0.086
	14	0	2.55	0.32
		1	2.59	0.31
	18	0	2.43	0.24
		1	2.70	0.27
		2	2.54	0.26
		3	2.87	0.19

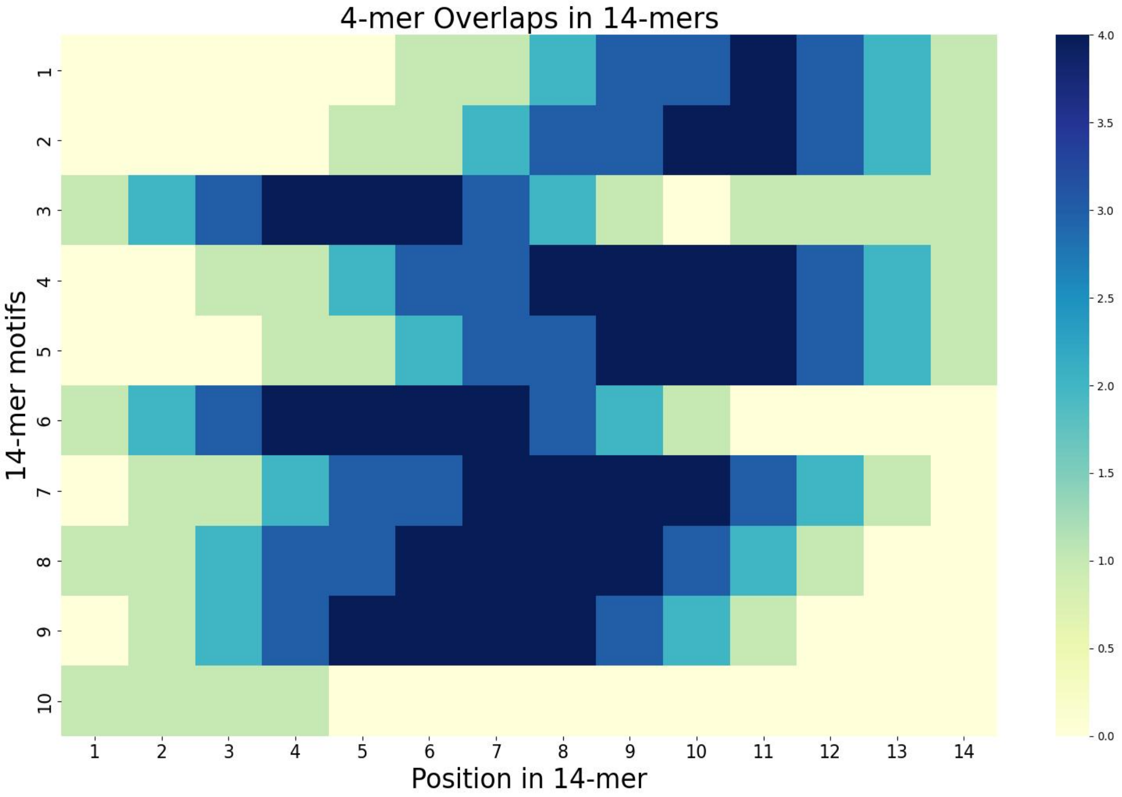

Figure S1: Shows the overlap of 4-mer motifs in 14-mers motifs extracted using frequency-based method. All 4-mer motifs were fully contained within the 14-mer motifs.

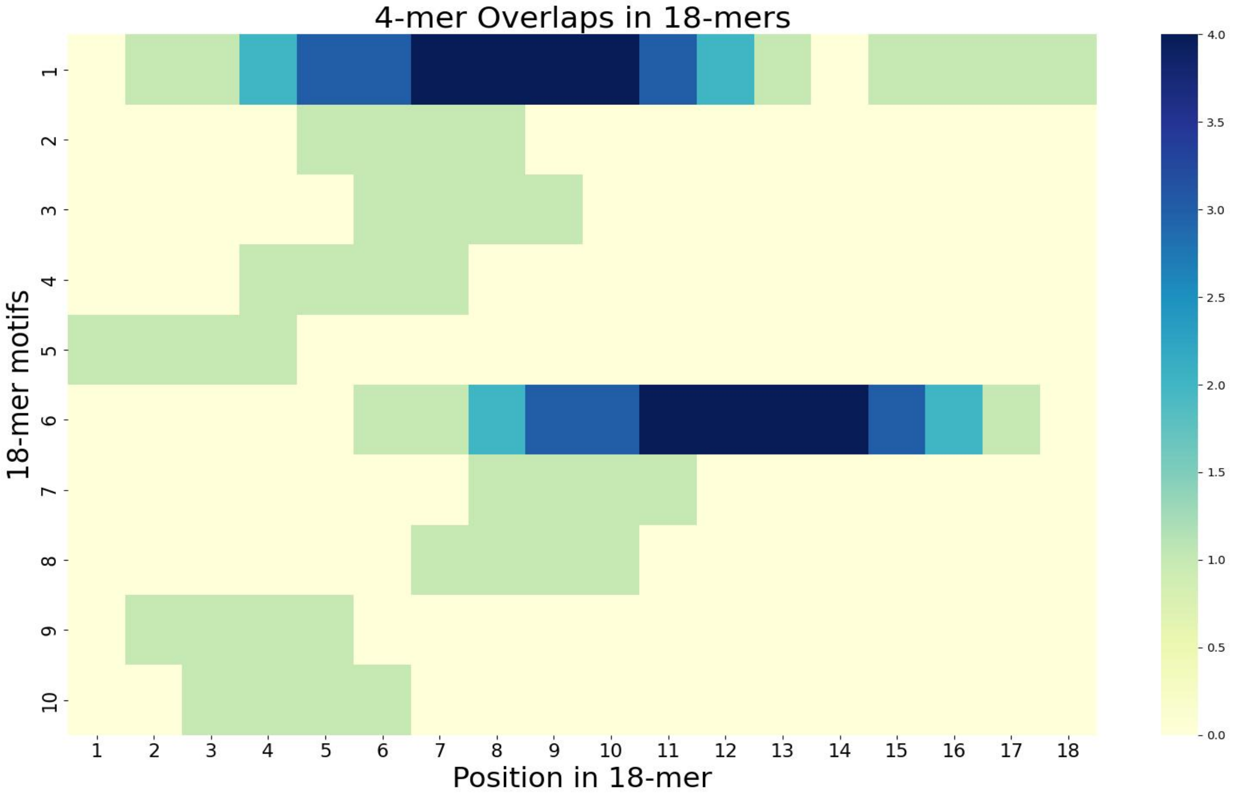

Figure S2: Shows the overlap of 4-mer motifs in 18-mers motifs extracted using frequency-based method. All 4-mer motifs were fully contained within the 18-mer motifs.

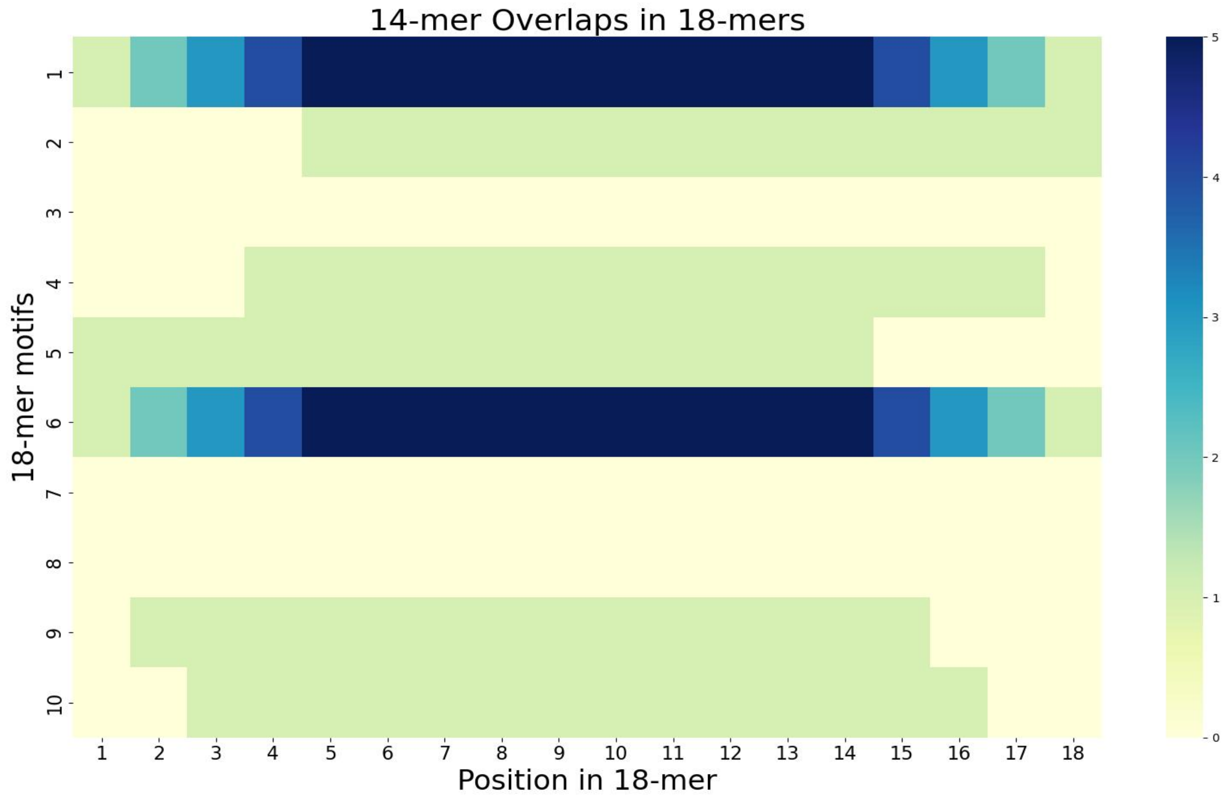

Figure S3: Shows the overlap of 14-mer motifs in 18-mers motifs extracted using frequency-based method. Most of 14-mers 7 out of 10 were found in the 18-mer motifs.

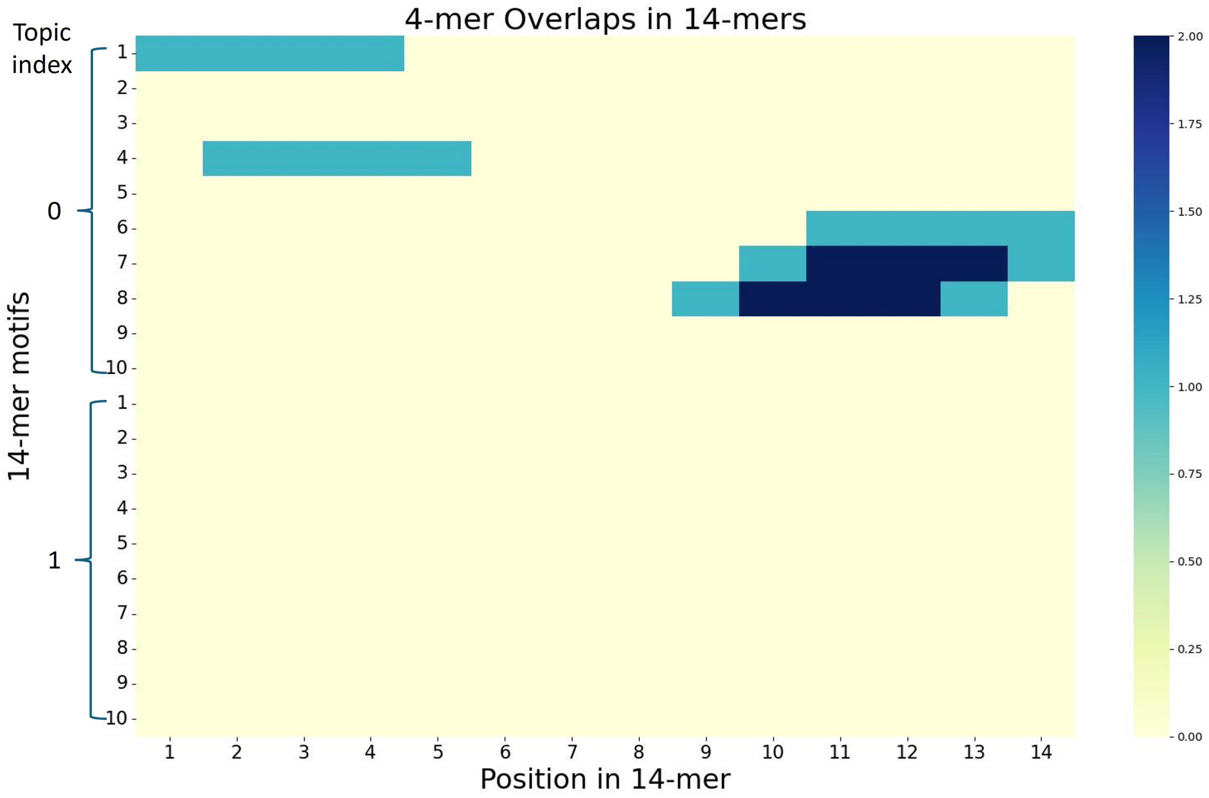

Figure S4: Shows the overlap of 4-mer motifs in 1-mers motifs extracted using LDA-based method. Only 3 out of 20 4-mers were found in the 14-mer motifs and these overlapped 4-mers were distributed across 5 (out of 20) of the 14-mers.

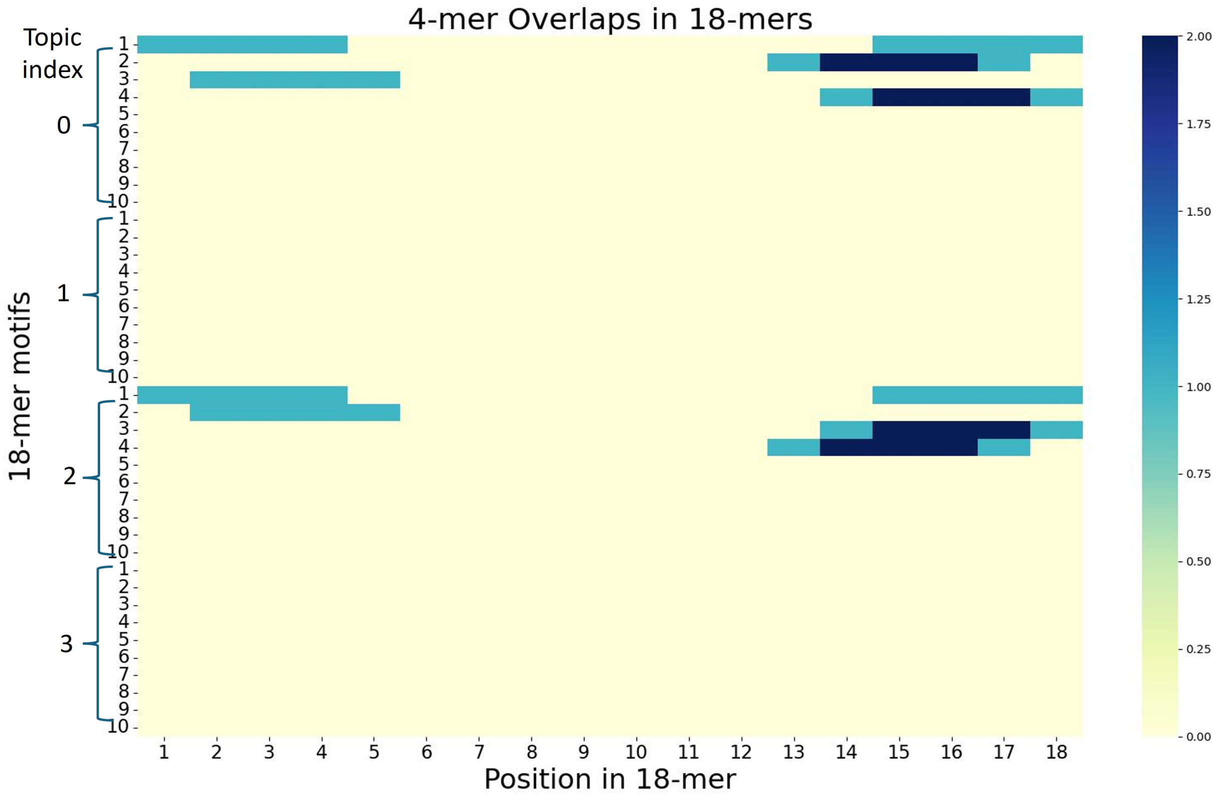

Figure S5: Shows the overlap of 4-mer motifs in 18-mers motifs extracted using LDA-based method. Only 8 out of 20 4-mers were found in the 18-mer motifs and these overlapped 14-mers were distributed across 8 (out of 40) of the 18-mers.

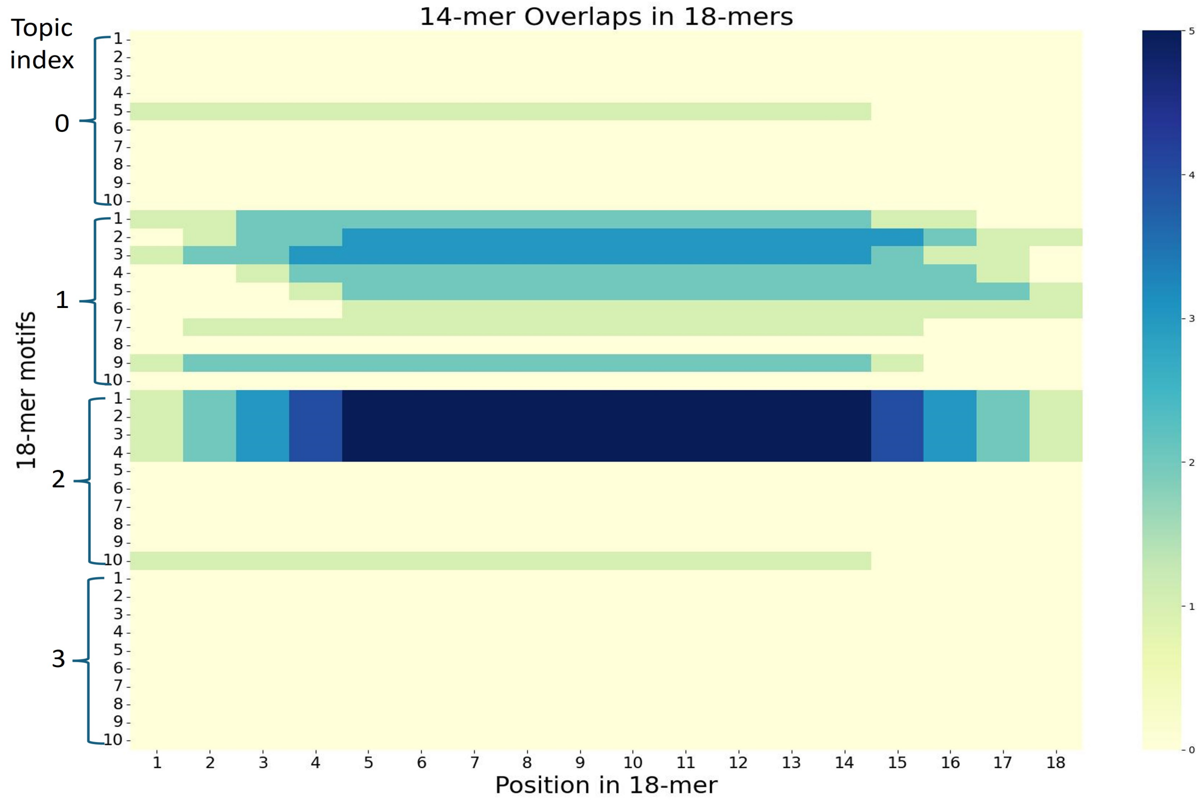

Figure S6: Shows the overlap of 14-mer motifs in 18-mers motifs extracted using LDA-based method. 17 out of 20 14-mers were found in the 18-mer motifs, but only 14 (out of 40) of the 18-mers contained them.

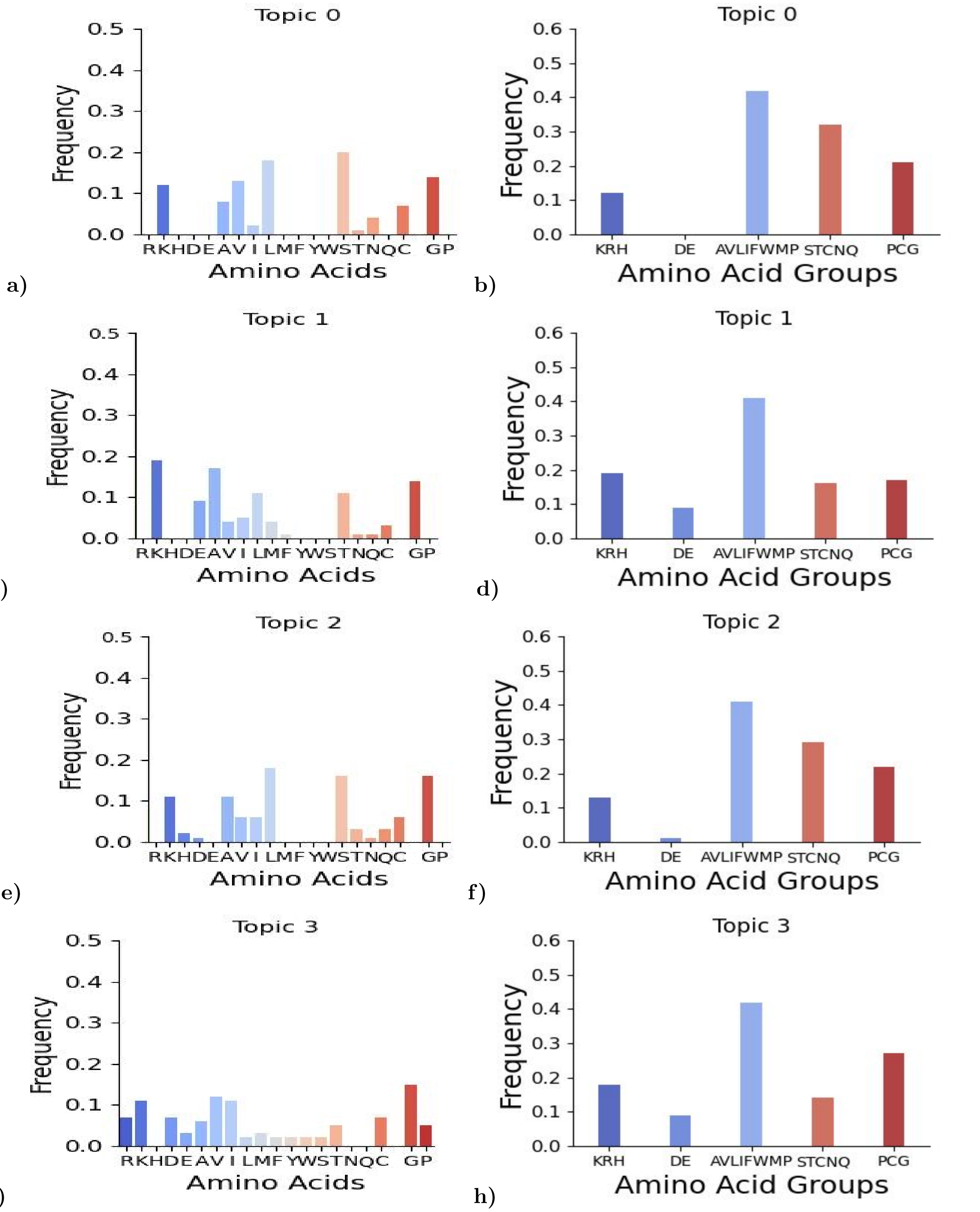

Figure S7: Amino acid compositions for the top ten LDA-derived 18-mer motifs associated with the *E. Coli* target. It depicts both individual amino acid compositions (panels a, c, e & g) for each topic and group-wise compositions (panels b, d, f & h) of topics 0-3.

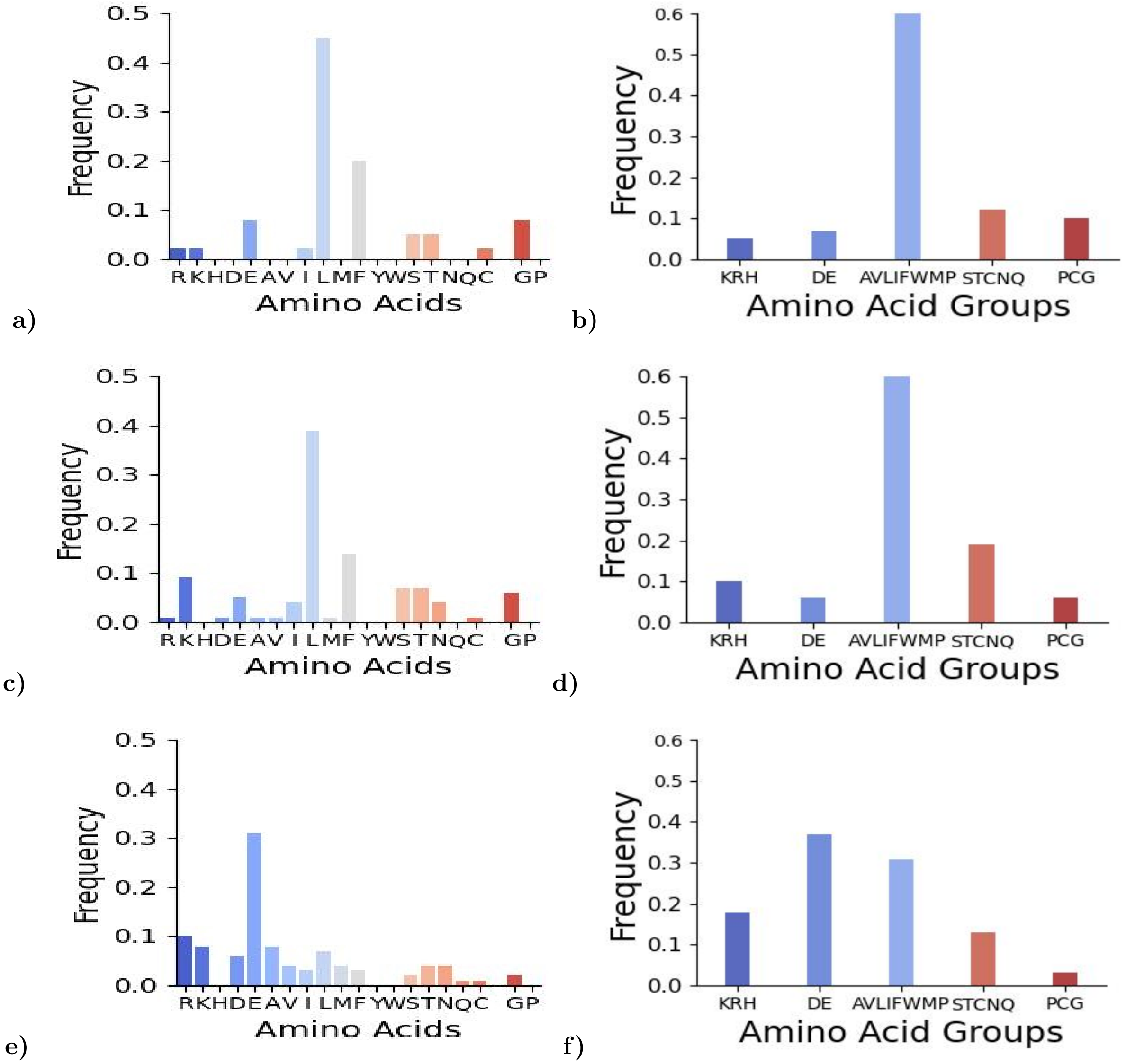

Figure S8: Amino acid compositions for the top ten frequency-based 4, 14, and 18-mer motifs associated with the *E. Coli* target. It depicts both individual amino acid compositions (panels a, c, & e) and group-wise compositions (panels b, d, & f). Note: There are no topic assignments to frequency-based analysis.

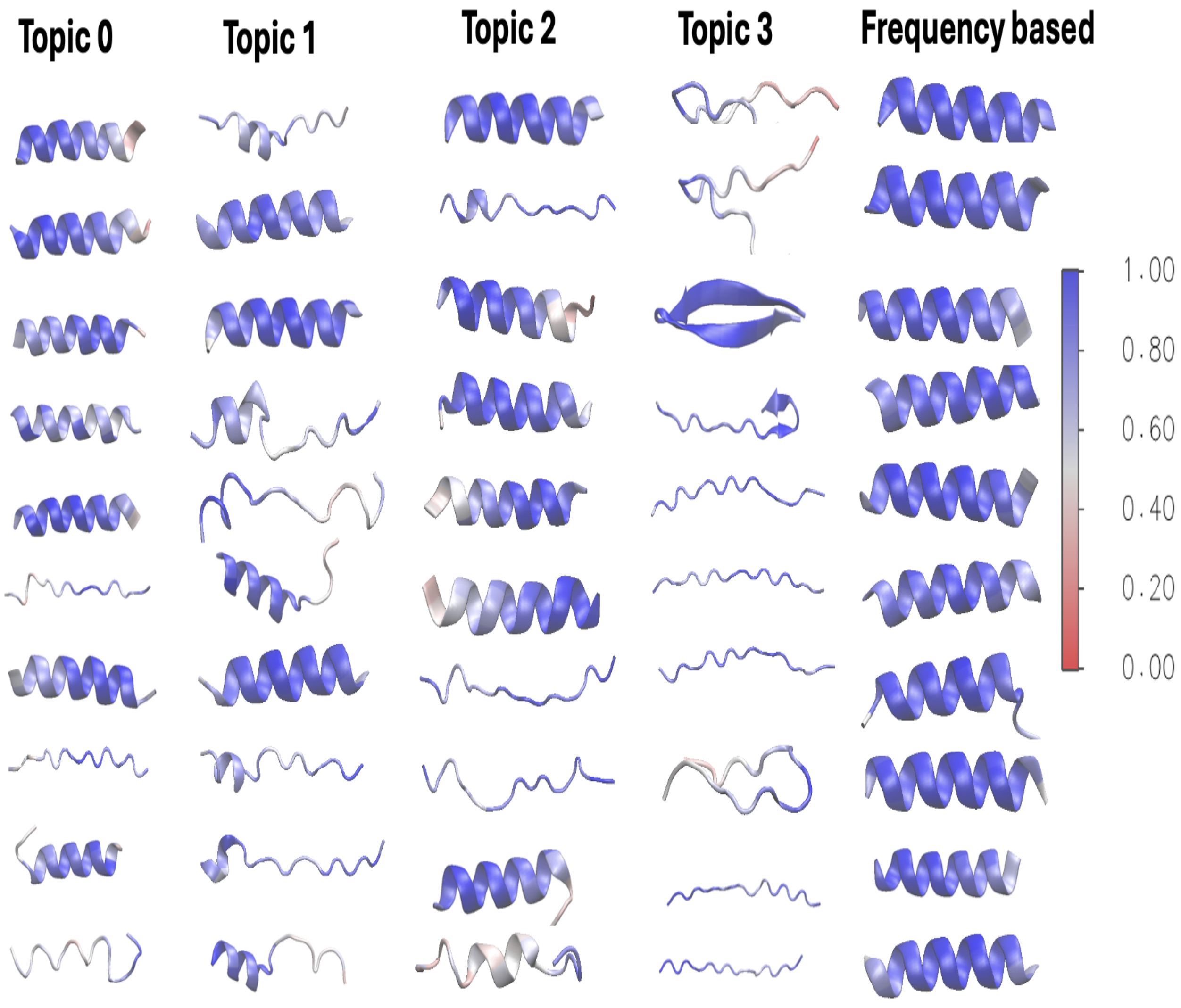

Figure S9: The analysis of the top ten LDA- and frequency-based 18-mer motifs shows that Topics 0-2 primarily feature helical structures, with three motifs in random coil structure. In contrast, Topic 3 includes only random coil structures. Similar to the 14-mer motifs, frequency-based motifs exhibit a more uniform structure overall. Additionally, the colorbar indicates the confidence levels of the predicted structures as determined by the ESMFold model.

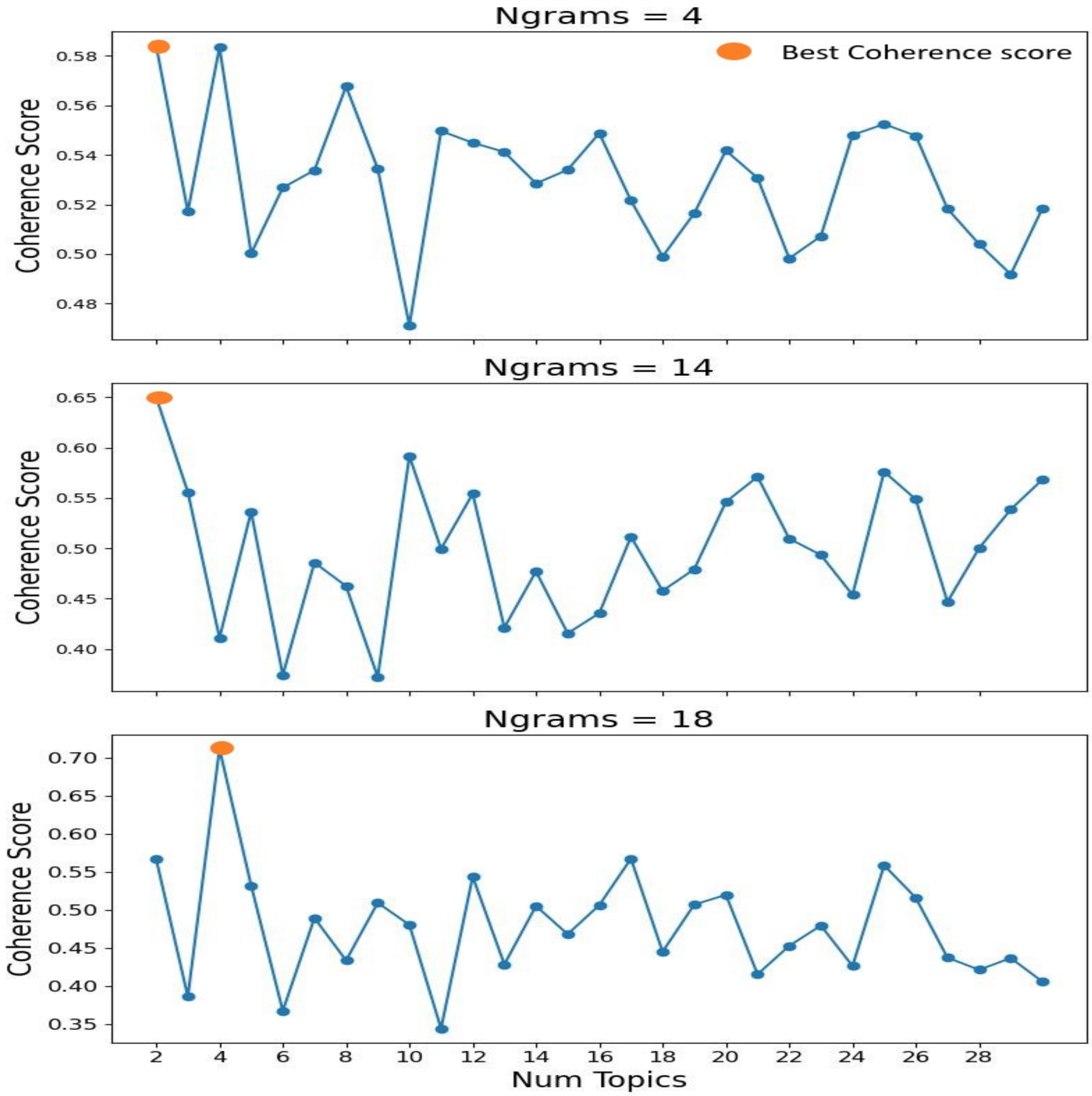

Figure S10: Shows coherence scores for k-mer lengths of 4, 14, and 18, with topic counts varying from 2 to 30. The maximum scores for k-mer lengths of 4, 14, and 18 are 2, 2, and 4, respectively.

References

[1] Nadin Shagaghi, Enzo A Palombo, Andrew HA Clayton, and Mrinal Bhave. Antimicrobial peptides: biochemical determinants of activity and biophysical techniques of elucidating their functionality. *World Journal of Microbiology and Biotechnology*, 34:1–13, 2018.

[2] Jack Kyte and Russell F Doolittle. A simple method for displaying the hydropathic character of a protein. *Journal of molecular biology*, 157(1):105–132, 1982.

[3] David Eisenberg, Robert M Weiss, and Thomas C Terwilliger. The hydrophobic moment detects periodicity in protein hydrophobicity. *Proceedings of the National Academy of Sciences*, 81(1):140–144, 1984.

[4] Zeming Lin, Halil Akin, Roshan Rao, Brian Hie, Zhongkai Zhu, Wenting Lu, Nikita Smetanin, Robert Verkuil, Ori Kabeli, Yaniv Shmueli, et al. Evolutionary-scale prediction of atomic-level protein structure with a language model. *Science*, 379(6637):1123–1130, 2023.

[5] Na Chen and Cheng Jiang. Antimicrobial peptides: Structure, mechanism, and modification. *European Journal of Medicinal Chemistry*, 255:115377, 2023.

[6] Beata Kowalska-Krochmal and Ruth Dudek-Wicher. The minimum inhibitory concentration of antibiotics: Methods, interpretation, clinical relevance. *Pathogens*, 10(2):165, 2021. PMID: 33557078, PMCID: PMC7913839.

[7] David Newman, Jey Han Lau, Karl Grieser, and Timothy Baldwin. Automatic evaluation of topic coherence. In *Human language technologies: The 2010 annual conference of the North American chapter of the association for computational linguistics*, pages 100–108, 2010.

[8] Michael Röder, Andreas Both, and Alexander Hinneburg. Exploring the space of topic coherence measures. In *Proceedings of the eighth ACM international conference on Web search and data mining*, pages 399–408, 2015.

[9] Fazlollah M Reza. *An introduction to information theory*. Courier Corporation, 1994.

[10] James V Stone. Information theory: A tutorial introduction, 2019.
